# Bioprospecting of Microalgae Isolated from the Adriatic Sea: Characterization of Biomass, Pigment, Lipid and Fatty Acid Composition, and Antioxidant and Antimicrobial Activity

**DOI:** 10.3390/molecules27041248

**Published:** 2022-02-12

**Authors:** Marina Grubišić, Božidar Šantek, Zoran Zorić, Zrinka Čošić, Ivna Vrana, Blaženka Gašparović, Rozelindra Čož-Rakovac, Mirela Ivančić Šantek

**Affiliations:** 1 Faculty of Food Technology and Biotechnology, University of Zagreb, 10000 Zagreb, Croatia; mgrubisic@pbf.hr (M.G.); bsantek@pbf.hr (B.Š.); zzoric@pbf.hr (Z.Z.); zcosic@pbf.hr (Z.Č.); 2 Laboratory for Marine and Atmospheric Biogeochemistry, Division for Marine and Environmental Research, Ruđer Bošković Institute, 10000 Zagreb, Croatia; ivna@irb.hr (I.V.); gaspar@irb.hr (B.G.); 3 Laboratory for Aquaculture Biotechnology, Division of Materials Chemistry, Ruđer Bošković Institute, 10000 Zagreb, Croatia; rrakovac@irb.hr; 4 Center of Excellence for Marine Bioprospecting (BioProCro), Ruđer Bošković Institute, 10000 Zagreb, Croatia

**Keywords:** bioprospecting, marine microalgae, biomass composition, fatty acids, growth rate, pigments, antioxidant activity, antimicrobial activity, glycolipids, phospholipids

## Abstract

Marine microalgae and cyanobacteria are sources of diverse bioactive compounds with potential biotechnological applications in food, feed, nutraceutical, pharmaceutical, cosmetic and biofuel industries. In this study, five microalgae, *Nitzschia* sp. S5, *Nanofrustulum shiloi* D1, *Picochlorum* sp. D3, *Tetraselmis* sp. Z3 and *Tetraselmis* sp. C6, and the cyanobacterium *Euhalothece* sp. C1 were isolated from the Adriatic Sea and characterized regarding their growth kinetics, biomass composition and specific products content (fatty acids, pigments, antioxidants, neutral and polar lipids). The strain *Picochlorum* sp. D3, showing the highest specific growth rate (0.009 h^−1^), had biomass productivity of 33.98 ± 0.02 mg L^−1^ day^−1^. Proteins were the most abundant macromolecule in the biomass (32.83–57.94%, g g^−1^). *Nanofrustulum shiloi* D1 contained significant amounts of neutral lipids (68.36%), while the biomass of *Picochlorum* sp. D3, *Tetraselmis* sp. Z3, *Tetraselmis* sp. C6 and *Euhalothece* sp. C1 was rich in glycolipids and phospholipids (75%). The lipids of all studied microalgae predominantly contained unsaturated fatty acids. Carotenoids were the most abundant pigments with the highest content of lutein and neoxanthin in representatives of Chlorophyta and fucoxanthin in strains belonging to the Bacillariophyta. All microalgal extracts showed antioxidant activity and antimicrobial activity against Gram-negative *E. coli* and *S. typhimurium* and Gram-positive *S. aureus*.

## 1. Introduction

Microalgae are major primary producers of organic matter in the marine environment, responsible for producing one half of global oxygen on the Earth [1,2]. The Adriatic Sea is one of the best-preserved biological reserves in the Mediterranean, whose microbial diversity is still largely unexplored [3]. Marine microalgae belong to five taxonomic divisions: (1) Chlorophyta (green algae); (2) Chrysophyta (golden-brown, diatoms, and yellow algae); (3) Pyrrhophyta (dinoflagellates); (4) Euglenophyta and (5) Cyanophyta (blue-green algae) [1,4]. Cyanophyta includes prokaryotic cyanobacteria, one of the most interesting sources of novel marine compounds [5]. The variety of primary and secondary metabolites synthesized by marine microalgae results from their adaptation to this unique habitat. These metabolites show numerous and different activities, including immune-modulating, anti-inflammatory, anti-cancer, antimicrobial, antioxidative activity, with potential use in the production of pharmaceuticals and nutraceuticals [1,2,5,6,7,8]. Besides, microalgae have many other commercial applications in the food and feed industries (e.g., animal feed, aquaculture, and human food), the production of beauty-related products, as biofertilizers, and in the production of advanced biofuels [9,10,11,12,13,14]. Microalgae have several advantages over land-based crops, including a fast growth rate, high lipid content, high biomass areal productivity, and can be grown on non-arable land such as marginal areas unsuitable for agricultural purposes [15,16]. One of the most important features of microalgae is the capacity to mitigate carbon dioxide, which makes production processes with microalgae more sustainable, economically viable and environmentally friendly. Furthermore, implementing those processes contributes to a sustainable energy supply, food security, creates new jobs, and stimulates economic growth [14,15,16]. The bioactive compounds that can be found in microalgae include polyphenols, carotenoids, polysaccharides, ω-3 fatty acids, and polyunsaturated fatty acids (PUFA) [17,18,19,20,21,22,23]. The lipid content in microalgae ranges between 20% to 70%. Microalgal lipids are generally considered a valuable source of polyunsaturated fatty acids (PUFAs), including ω-3 (eicosapentaenoic acid (C20:5), docosahexaenoic acid (C22:6), α-linolenic (C18:3 (ω-3)) and ω-6 (linoleic (C18:2), and γ-linolenic (C18:3 (ω-6)) fatty acids [22,24]. Diatoms, microalgae belonging to the division Bacillariophyceae, are an important source of eicosapentaenoic acid with 15% to 30% in total lipids [18,23]. These fatty acids are used to treat cancer, Alzheimer’s, modulatory vascular resistance, atherosclerosis and infant malnutrition [25]. Furthermore, microalgal biomass is a rich source of carbohydrates, proteins, with all essential amino acids, enzymes, and fibres and, therefore, is used as a supplement to improve the nutritional value of food [26,27,28]. It is also a source of many vitamins (A, C, B1, B2, B6, and niacin), minerals (potassium, iron, magnesium, calcium) and pigments [29]. A number of studies demonstrate the high antioxidant activity of some microalgal and cyanobacterial species [2,21,30,31,32,33,34,35,36,37,38,39]. With the global trend of using natural antioxidants instead of synthetic ones, microalgae have also been considered an alternative source of natural antioxidants. Flavonoids, phenols, fatty acids, aromatic amino acids, and other non-classified compounds contribute to the total antioxidant capacity of microalgae. The importance of phenolic compounds to the total antioxidant activity of microalgae is still discussed, but several studies indicate their significant contribution. Goiris et al. [32] studied 32 microalgae species for antioxidant capacity in relation to their phenolic and carotenoid content. Results suggest that, besides carotenoids, phenolic compounds significantly contribute to the antioxidant capacity of microalgal biomass. Microalgal extracts have high antimicrobial activity against different pathogenic microorganisms, including *Staphylococcus aureus*, *Bacillus cereus*, *Listeria monocytogenes*, *Escherichia coli*, *Salmonella* spp., *Aspergillus niger*, and *Aspergillus flavus* [39,40,41]. The antimicrobial potential is also affected by all these major bioactive constituents of microalgal biomass including proteins, polysaccharides, polyunsaturated fatty acids (PUFAs), especially eicosapentaenoic acid (C20:5) and docosahexaenoic acid (C22:6), amino acids, and antioxidants (polyphenols, flavonoids, carotenoids).

The focus of the project Bioprospecting of the Adriatic Sea was to isolate marine microorganisms, including heterotrophic and phototrophic, with potential commercial applications. Water samples were collected from the different regions of the Adriatic Sea. After the initial selection, the growth kinetics of the fast-growing strains were further analyzed. Finally, five microalgae (*Nitzschia* sp. S5, *Nanofrustulum shiloi* D1, *Picochlorum* sp. D3, *Tetraselmis* sp. Z3 and *Tetraselmis* sp. C6) and one cyanobacterium (*Euhalothece* sp. C1) were chosen based on the characteristics of strains described in the literature. The *Tetraselmis* strains are the most promising candidates for commercial biodiesel production since they efficiently accumulate lipids, even during their exponential growth phase, which makes these strains suitable for continuous cultivation. Due to lower costs, continuous cultivation is preferred over batch cultivation in large-scale production. Furthermore, *Tetraselmis* strains were successfully cultivated in open ponds under outdoor conditions in high saline water over a long period [42]. The *Picochlorum* genus recently has received much attention due to the high growth rate (0.9 d^−1^), high lipid accumulation rate (20–58%), tolerance to high NaCl concentrations (growth rate of 0.65 d^−1^ at 70 ppm NaCl), growth at elevated temperatures (from 35 to 40 °C), and high-light intensities [43,44,45]. The marine diatoms *Nitzschia* sp. and *N. shiloi* have the ability to accumulate long-chain polyunsaturated fatty acids such as ω-3 (eicosapentaenoic acid) and fucoxanthin under abiotic stress, such as nitrogen and silicon limitation, salinity stress or light intensity. Relatively high growth rates (0.21–0.37 d^−1^) with a significant content of lipids (up to 37%) rich in polyunsaturated fatty acids make these diatoms an interesting food and feed production platform [46,47,48,49,50]. Cyanobacteria have certain advantages over eukaryotic green microalgae and diatoms. Most of the microalgae prefer neutral to a slightly basic pH at which CO_2_ absorption is low. Absorption of CO_2_ decreases the pH of the growth medium and thus reduces the growth rate. The extremely salt-tolerant cyanobacterium *Euhalothece* sp. favours growth at high alkalinity conditions (pH = 9.8–10) at which absorption of CO_2_ is significantly higher and fluctuations in pH due to CO_2_ absorption are negligible [51,52]. Since the *Euhalothece* strain is capable of fixing atmospheric nitrogen, using this cyanobacterium in biofuel production could reduce the production costs by circumventing the large nitrogen requirements for cell growth [53]. The growth of selected strains was characterized by a specific growth rate, cell number and overall productivity. Microalgal biomass was analyzed for macromolecular (proteins, carbohydrates, lipids), pigments, carbohydrates, fatty acids, and lipid classes composition. Furthermore, antioxidant potential and the antimicrobial activity against chosen bacterial, yeast and fungal strain were determined.

## 2. Results and Discussion

### 2.1. Microalgae Growth

**Figure 1:**
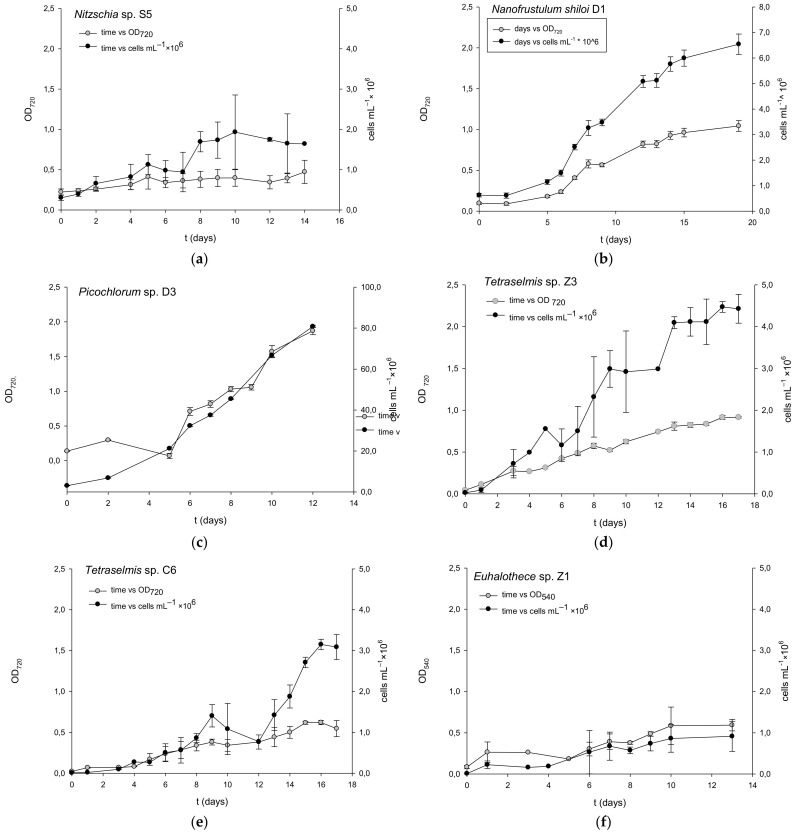
Growth curves for (**a**) *Nitzschia* sp. S5; (**b**) *Nanofrustulum shiloi* D1; (**c**) *Picochlorum* sp. D3; (**d**) *Tetraselmis* sp. Z3; (**e**) *Tetraselmis* sp. C6; (**f**) *Euhalothece* sp. C1.

Microalgae growth was determined by measuring optical density and the number of cells per mL of culture (Figure 1). The growth curves had characteristic shape observed for single-cell microorganisms, starting with an exponential phase that lasted 10–15 days, depending on the microalgal strain. Cultivations were stopped after the growth rate began to decrease and cells entered the stationary phase. The growth rate is a critical parameter for selecting the production strain since a high growth rate enables high productivity, reduces contamination risk, and decreases the cultivation time. The growth rates were calculated from the slope of semi-logarithmic plots of cell number (cells per mL) versus time. *Picochlorum* sp. D3 and *Nitzschia* sp. S5 had the highest growth rate of 0.01 and 0.009 h^−1^, respectively (Table 1). Observed growth rates of studied green microalgae and diatoms were much lower than reported values [54,55,56]. Cyanobacteria *Euhalothece* C1 grew slower than both diatoms (0.00375 h^−1^) and faster than the *Tetraselmis* strains Z3 and C6. A moderately higher growth rate (0.0046 h^−1^) was reported by Yang et al. for *Euhalothece* sp. Z-M001 growing under optimized conditions in the presence of 3% NaCl at 28 °C [57]. Most of the cultivations in reported studies were conducted under optimized conditions (temperature, pH, growth, medium composition, light) for specific strains using CO_2_ enriched air that provides higher growth rates and biomass yields. In contrast, our study aimed to characterize and compare the growth of selected microalgae in a standard growth medium for microalgae (Guillard’s f/2 medium) under unoptimized conditions. In order to improve productivity and biomass yield, making selected strains relevant for application in food, feed, pharmaceutical and biofuels production platforms, further efforts are needed in optimizing growth conditions and nutrient composition.

The highest cell concentration of 0.45 and 0.48 g L^−1^ were obtained by *Tetraselmis* strains C6 and Z3 in the early stationary phase. Similar maximal concentrations of cell biomass (0.35–0.46 g L^−1^) were reported for *Tetraselmis tetrathele*, *Tetraselmis striata*, *Tetraselmis chuii*, and *Tetraselmis gracili* growing under the same growth conditions [58]. The biomass concentration of *Picochlorum* sp. D3 obtained in this study was moderately higher than that of *Picochlorum atomus*, cultivated under similar growth conditions using fluorescent light (0.27 g L^−1^) [59]. Biomass concentrations of diatoms and cyanobacteria were lower than those reported in the literature. Under optimized conditions (120 g L^−1^ of NaCl, T = 35 °C), the biomass of *Euhalothece* sp. nov reached 2–4 g L^−1^ [53]. Despite relatively high growth rates, the lowest cell concentrations were obtained by diatoms (0.1 g L^−1^). Their biomass concentration was comparable to that in the study of Chu et al. (1996) [56]. Diatom *Nitzschia inconspicua* grew in an optimized growth medium at a rate of 0.33 h^−1^, reaching a maximal 150 mg L^−1^ of biomass. The maximum cell density of 8.08 × 10^7^ cell mL^−1^ was obtained on the 12th day of cultivation by *Picochlorum* sp. D3, while the cell densities of other strains were significantly lower. Since the cell yield depends not only on the growth rate but also on cell number at the beginning of the exponential phase, poor cell density could be attributed to a low cell density of inoculum. The initial cell concentration of *Picochlorum* sp. D3 was 3.03 × 10^6^ cell mL^−1^, while the initial concentration of other strains was several times lower, i.e., between 1.0 × 10^4^ and 6.27 × 10^5^ cell mL^−1^.

### 2.2. Biomass Composition

**Figure 2:**
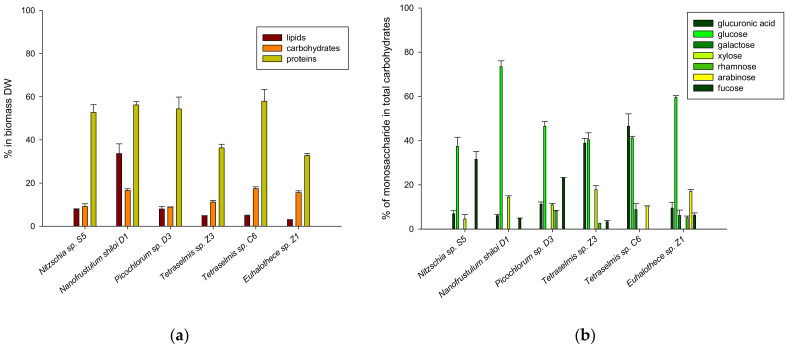
(**a**) Macromolecular composition of microalgal biomass; and (**b**) monosaccharides composition of carbohydrates.

The macromolecular composition of selected microalgae is presented in Figure 2a. Under nitrogen repletion conditions, proteins were the most abundant macromolecule in the biomass of all three classes of microalgae. *Tetraselmis* C6 had the highest protein content (62.86%), followed by diatoms *Nitzschia* sp. S5 (59.28%) and *Nanofrustulum shiloi* D1 (56.22%) and green microalgae *Picochlorum* sp. D3 (52.07%). Low protein content was observed in *Tetraselmis* sp. Z3 (36.32%) and cyanobacteria *Euhalothece* C1 (32.83%). The protein content in *Nitzschia* sp. S5, *Nanofrustulum shiloi* D1 and *Picochlorum* sp. D3 was comparable with the literature data [55,60,61]. Most cyanobacteria have high protein content that often exceeds 60% of dry cell biomass [62,63,64,65,66]. However, the protein content of *Euhalothece* sp. C1 was similar to cyanobacteria *Synechocystis aquatilis* SAG 90.79 [63]. Although the protein content in two *Tetraselmis* strains remarkably differed, their values were not unusual compared to the literature data. Most of the reported values for the *Tetraselims* strains were below 41% of dry cell biomass [28,55,63]. However, Kassim et al. (2014) determined 63.04% of the protein in biomass of *T. suecica* grew until the late exponential phase [65]. Lipids and carbohydrates were present in lower quantities in all selected microalgae. The biomass of *Nanofrustulum shiloi* D1 contained the highest lipid content (33.65%, g g^−1^), followed by *Nitzschia* sp. S5 (7.76%, g g^−1^) and *Picochlorum* sp. D3 (7.98%, g g^−1^), *Tetraselmis* sp. Z3 (4.87%, g g^−1^) and *Tetraselmis* sp. C6 (4.77%, g g^−1^), and *Euhalothece* sp. C1 (2.98%, g g^−1^). *Nanofrustulum shiloi* D1 (17.14%) and *Tetraselmis* sp. C6 (17.31%) had the highest carbohydrate content, followed by *Euhalothece* sp. C1 (15.28%). The protein content in *Euhalothece* sp. C1 was comparable with the literature data for cyanobacteria, while the lipid content was considerably lower [66]. The protein and lipid content of green microalgae and diatoms were similar to the reported data [28,55,67,68]. A low lipid and carbohydrate content in the cell biomass was expected since the cells were harvested in the late exponential phase or the beginning of the stationary phase. Specific macronutrients (e.g., nitrogen, phosphorus or silicon) were probably still available in growth media and thus, the carbon source was used for cell growth and protein synthesis [69,70]. Under stressful conditions, carbon flux is redirected towards the production of energy reserves. Growth under nutrient limitation (e.g., nitrogen, phosphorus, and silicon) in the presence of an excess carbon source enables the accumulation of carbohydrates and/or lipids [71,72,73,74,75]. Due to a high protein content, all studied strains of microalgae and cyanobacterium could be used as food and feed supplements. Furthermore, the high lipid level makes *Nanofrustulum shiloi* D1 an interesting source of renewable and sustainable feedstock for biodiesel production.

Carbohydrates have a specific physiological function in the microalgal cell. Based on their role, carbohydrates are divided into three groups: storage polysaccharides, cell-wall related polysaccharides and exopolysaccharides [76,77]. Cyanobacteria accumulate glycogen as intracellular storage carbohydrates, whereas eukaryotic microalgae preferably accumulate starch or β-glucans [77,78]. The carbohydrate composition differs between the species and can be affected by the growth conditions. Marine carbohydrates are mainly composed of neutral monosaccharides (including glucose, galactose, xylose, mannose, rhamnose, arabinose, fucose and ribose), amino sugars (glucosamine) and uronic acids (galacturonic and glucuronic) [76,77]. The composition of monosaccharides in the carbohydrate of studied microalgal species is presented in Figure 2b. Consistent with the literature data, glucose was the principal monosaccharide in all studied microalgae except for *Tetraselmis* sp. C6. Instead of glucose, this strain contained glucuronic acid as the most dominant monosaccharide (46.53%) [28,79,80,81]. Diatom *Nanofrustulum shiloi* D1 (73.09%) had the highest glucose content similar to diatom *Thalassiosira pseudonana* (82.4%) [80]. Both *Tetraselmis* strains had an unusually high glucuronic acid content compared with the literature data [76]. The second most abundant monosaccharide in *Nitzschia* sp. S5 and *Picochlorum* sp. D3 was fucose (31.56 and 23.09%, respectively), and it was present at lower levels (2.63–8.21%) in other studied microalgae except for *Tetraselmis* sp. C6. The fucose level in this study was significantly higher than reported previously. Brown (1991) reported fucose levels at 2.8–14.3% in six diatoms, *Chaetoceros calcitrans*, *Chaetoceros gracilis*, *Nitzschia ciosterium*, *Phaeodactylum tricornutum*, *Skeletonema costatum* and *Thalassiosira pseudonana* [80]. Unlike *Picochlorum* sp. D3, reported values of the fucose content in green microalgae, *Tetraselmis chui* and *Nannochloris* sp., are significantly lower [76]. Several other monosaccharides detected at a lower level included galactose (6.20–8.78%), xylose (4.54–17.82%), rhamnose (2.63–8.21%) and arabinose (10.49–17.09%). Arabinose and galactose were detected only in *Tetraselmis* sp. C6 and *Euhalothece* sp. C1, while xylose was present in diatoms and green algae strains except for *Tetraselmis* sp. C6. Carbohydrate composition from only two of the six studied microalgal species was reported previously. The results obtained in this study are quantitatively comparable to the reported values for similar strains belonging to the same class [76,80].

### 2.3. Fatty Acid Composition

Microalgae accumulate storage compounds under stress conditions such as nutrient/s depletion in the presence of a carbon source. Since the cultures were grown until the early stationary phase, an effect of growth limitation and significant accumulation of storage compounds, including lipids and carbohydrates, were not expected [75,82,83]. Under these conditions, most fatty acids were probably incorporated into the cell membranes. The fatty acid composition of isolated microalgae is summarized in Table 2. Palmitic acid (C16:0) was one of the most abundant fatty acids in all studied microalgae (23.19–33.25%). Fatty acid profiles significantly varied between different microalgal classes and strains. The most common fatty acids in diatoms are myristic acid (14:0), palmitic acid (C16:0), palmitoleic acid (C16:1), docosahexaenoic acid (DHA, C22:6), and eicosapentaenoic acid (EPA, C20:5) [23]. The number of double bonds in fatty acid chains is usually two or three, and rarely more than six. The most abundant fatty acids in both studied diatoms, palmitoleic acid (C16:1) and palmitic acid (C16:0), made more than 70% of total fatty acids in the lipid extract. Diatoms contain significant amounts of ω-3 and 6 fatty acids, including alpha-linolenic acid (ALA, C18:3), eicosapentaenoic acid (EPA, C20:5), docosahexaenoic acid (DHA, C22:6), arachidonic acid (ARA, C20:4) and linoleic acid (C18:2). *Nanofrustulum shiloi* D1 contained a significant amount of arachidonic acid (ARA, C20:4; 9.29%), followed by eicosapentaenoic acid (EPA, C20:5; 6.79%) and alpha-linolenic acid (ALA, C18:2; 1.88%). *Nitzschia* sp. S5 had a fatty acid profile comparable to *Nanofrustulum shiloi* D1, with a higher myristic acid (C14:0) content (10.59% versus 0.66%, respectively), lower eicosapentaenoic acid (EPA, C20:5; 3.48%) and an absence of arachidonic acid. Odd-chain fatty acids, margaric (C17:0) and heptadecanoic (C17:1) acid were also present in lipids, but at lower levels. Odd chain fatty acids are frequently used as biomarkers for bacterial contamination of microalgae cultures [84]. In microalgae, they can also be found, but in the content that usually does not exceed 5% [85]. On the other hand, Ghazala and Shameele found a significantly higher amount of odd chain FA (up to 52.2% of all FAs) in freshwater green microalgae [86]. The intake of odd FAs has a positive effect on humane health, e.g., reducing the risk of developing multiple sclerosis [87] and an anti-carcinogenic effect on cancer cells [88], so isolated *Picochlorum* sp. D3 and *Euhalothece* sp. C1 from the Adriatic Sea have the potential to become a food supplement with health benefits for humans. The fatty acid profile and content of most dominant fatty acids were in accordance with those reported for marine diatoms [23,89]. Most of the cyanobacteria have a high relative content of palmitic acid (C16:0), followed by palmitoleic (C16:1), stearic (C18:0), and unsaturated C18 fatty acids (C18:1, C18:2 and C18:3) [90,91]. However, the most dominant fatty acids in *Euhalothece* sp. C1 was odd-chain margaric acid (C17:0; 48.79%), which was also found in some heterocystous cyanobacteria, but at a significantly lower levels (1.57–5.84%) [90]. However, the composition of fatty acids of the same microalgae species can significantly vary depending on the cultivation conditions and growth phase [55,92,93]. The relative content of palmitic (C16:0, 28.40%) and palmitoleic acid (C16:1, 1.36%) was comparable to the cyanobacterium *Nostoc punctiforme*, while the level of oleic (C18:1, 0.84%), and linoleic acid (C18:2 trans 2.54%, C18:2 cis 3.99%) was significantly lower [55,93]. Three green microalgae had similar fatty acid profiles with a high relative content of palmitic (C16:0, 28.14–33.25%), oleic (C18:1, 2.92–16.56%), linoleic (C18:2, 8.5–33.3%) and alpha-linolenic (C18:3, 11.59–22.52%) fatty acid. Both *Tetraselmis* strains had a similar relative content of eicosapentaenoic acid (EPA, C20:5; 8.85 and 7.01%), which was not detected in *Picochlorum* sp. D3. Furthermore, margaric (C17:0, 22.23%) was also found in *Picochlorum* sp. D3 at high levels. Our results were inconsistent with the literature data for different strains of *Picochlorum* [55,94]. However, the content of this fatty acid in *Nannochloropsis* and *Tetraselmis* strains could reach up to 12% [55,95].

Due to the specific fatty acid profile, studied microalgae could be used in food, biofuels and nutraceuticals production. *Tetraselmis* sp. Z3 contained a high level of polyunsaturated fatty acids, including ω-3 fatty acid eicosapentaenoic acid (EPA, C20:5), docosahexaenoic acid (DHA, C22:6) and alpha-linolenic (C18:3), which can be used as a fish oil replacement. Polyunsaturated fatty acids, especially ω-fatty acids like eicosapentaenoic acid (EPA, C20:5) and docosahexaenoic acid (DHA, C22:6) are essential for human health, especially for infant development, prevention of chronic disease and reducing the risk of heart diseases [96,97]. Four microalgae (*Nanofrustulum shiloi* D1, *Picochlorum* sp. D3, *Tetraselmis* sp. Z3 and *Tetraselmis* sp. C6) have the potential for use as a food supplement enriched with polyunsaturated fatty acids. The unusually high content of C17:0 in *Picochlorum* sp. D3 and *Euhalothece* sp. C1 makes these strains an interesting platform for producing odd chain fatty acids. Odd chain fatty acids, including C15:0 and C17:0, found in studied microalgae have beneficial effects on human health. The intake of these fatty acids lowers the risk of multiple sclerosis, coronary heart disease, and type II diabetes [98,99,100,101]. Furthermore, odd chain fatty acids and their derivates are used as precursors and building blocks in producing chemicals, biocides and flavours [102]. The fatty acid profile of *Nitzschia* sp. S5 and *Euhalothece* sp. C1 lipids enriched in the C16–C18 fatty acids with low polyunsaturated fatty acid content makes them suitable for biodiesel production.

### 2.4. Lipid Class Composition

All three classes of microalgae differed in lipid classes composition (Table 3). Neutral lipids comprised mono, di and triacylglycerols, free fatty acids, sterols and steryl esters. Polar lipids included phospholipids (phosphatidylglycerol, phosphatidylethanolamine and phosphatidylcholine) and glycolipids (monogalactosyldiacylglycerols, digalactosyldiacylglycerols and sulfoquinovosyldiacylglycerols). Green microalgae and cyanobacteria contained the least neutral lipids (15.22–18.58%) and the highest amount of polar lipids (74.59–78.33%). On the contrary, diatoms had more neutral lipids (46.60–68.36%) and less polar lipids (29.03–49.33%). Relatively low amounts of triacylglycerols were observed in all studied species since the cells were harvested in the late exponential or early stationary phase of growth when triacylglycerols are present in low amounts [75,82,83]. 

Free fatty acids were the most dominant fraction of neutral lipids in all three microalgal classes. Green microalgae, *Tetraselmis* sp. Z3 and *Tetraselmis* sp. C6, were found to contain the lowest amount of fatty acids (5.80–10.25%), followed by cyanobacterium with 15.23%. The content of free fatty acids in these strains was significantly lower than those reported for green microalgae [103] and cyanobacteria [104]. Improper storage and handling of the samples prior to analysis may significantly influence accuracy. Thus, higher levels of fatty acids reported in the literature might result from lipid degradation during the storage or processing of the samples [103,105]. On the contrary, free fatty acids in diatoms were higher than in the literature data despite the proper handling of samples during analysis [106,107,108,109]. High free fatty acid content could be ascribed to triacylglycerol hydrolysis by endogenous lipases. These enzymes provide a carbon source and energy through the hydrolysis of triacylglycerol stored during the photosynthetic active light phase during the dark period of growth [110]. Since the analyzed cell biomass in this work was harvested after a dark period of growth, the activity of lipases is expected to be relatively high, especially at the end of the stationary phase when cell growth is limited by carbon source and/or light [110]. Microalgae produce an array of sterol compounds that serve as regulators of the membrane’s fluidity and permeability, signaling molecules and modulators of membrane-bound protein functions [111,112]. In most of the microalgae, sterols are present in free form (i.e., non-esterified), although they can also exist as conjugates (e.g., steryl esters, steryl glycosides and acyl steryl glycosides) [113]. Steryl esters dominated over sterols in diatoms *Nanofrustulum shiloi* D1 and *Nitzschia* sp. S5 (5.71 and 1.71% versus 2.03 and 1.30%, respectively) as well as in the cyanobacterium *Euhalothece* sp. C1. However, the synthesis of sterols has been a matter of dispute among phycologists, and most of the conducted research suggests that cyanobacteria do not synthesize sterols [114].

In this study, significant variations in neutral and polar lipid content were observed between major microalgae classes, even between the strains. The neutral to polar lipids ratio varied in diatoms (for *Nanofrustulum shiloi* D1 2.35, and *Nitzschia* sp. S5 0.94), while in cyanobacteria and green microalgae, the ratio was almost constant (0.20–0.25). The ratio changes depends on the growth phase but also on cultivation conditions (light-dark cycle, light intensity) and growth media composition [115]. Polar lipids contributed to more than 75% of the total lipids in three studied green microalgae and cyanobacterium with comparable amounts of glycolipids and phospholipids. The major components of chloroplast lipids in microalgae are glycolipids (monogalactosyldiacylglycerol, digalactosyldiacylglycerol and sulfoquinovosyldiacylglycerol) and phospholipids (phosphatidylglycerol, phosphatidylcholine, phosphatidylethanolamine, phosphatidylserine, and phosphatidylinositol) [103]. The most dominant polar lipids in all studied microalgae were monogalactosyldiacylglycerol (4.66–22.48%), sulfoquinovosyldiacylglycerol (4.02–14.89%), phosphatidylglycerol (10.00–36.83%) and phosphatidylethanolamine (5.25–19.88%). Unlike the other studied microalgae, a lipid extract from *Picochlorum* sp. D3 contained a significant amount of phosphatidylcholine (8.08%). *Tetraselmis* sp. C6, *Tetraselmis* sp. Z3 and *Euhalothece* sp. C1 had similar content of monogalactosyldiacylglycerol (4.66–9.29%), sulfoquinovosyldiacylglycerol (11.78–14.89%), phosphatidylglycerol (36.08–36.83%), and phosphatidylethanolamine (13.26–19.88%). However, *Picochlorum* sp. D3 contained a significantly higher amount of monogalactosyldiacylglycerol (22.48%) and a much lower phosphatidylglycerol (21.93%). The observed content of phospholipids and glycolipids were significantly higher than reported for green marine microalgae *Nannochloropsis* sp. and freshwater microalgae *Chlamydomonas reinhardtii*, *Chlorella vulgaris* and *Scenedesmus* sp. as well as traustrochytrid, *Schizochytrium limacinum* [103]. Furthermore, the profile of polar lipids in green microalgae and cyanobacterium significantly differed from those obtained in this study [103,116,117,118]. However, the lipid profile may significantly vary even within the same genus [119]. As microalgae in this study were isolated from the Adriatic Sea, inconsistency with the literature data is not surprising. The most dominant lipid fractions in cyanobacterial strains are monogalactosyldiacylglycerol (~40%) and digalactosyldiacylglycerol (~32%), followed by sulfoquinovosyldiacylglycerol (~15%), and phosphatidylglycerol (~13%) [116,117,118]. The high level of phospholipids with several ω-3 and 6 fatty acids (Table 2.) makes studied diatoms and green microalgae a potentially interesting supplement for food and feed. Several studies have shown that the type of fatty acid ester used as the supplement may affect the absorption, distribution and tissue uptake of these important fatty acids. Phospholipids are more suitable form compared with triacylglycerol. Furthermore, glycolipids and phospholipids from marine algae have additional biological activity [120]. Antimicrobial, antiviral, antiinflammatory and immunotropic properties of marine macrophyte extracts are related to glycolipids and phospholipids [121,122,123]. All studied microalgae had a low hydrocarbon content (1.53–3.77%) in accordance with reported values [103,124,125].

### 2.5. Pigment Content and Composition

Pigments are high-value products with a broad application, mainly in the food, cosmetic and pharmaceutical industries. Additionally, pigments contribute to antioxidant and antimicrobial activity in microalgae biomass. The content of pigments in six different microalgae species was monitored by HPLC-PDA analysis. Six carotenoids and two chlorophylls, namely neoxanthin (and its four derivates), fucoxanthin and one fucoxanthin derivate, canthaxanthin and its two derivates, lutein and two derivates, α-carotene, β-carotene, chlorophyll-a and six derivates, and chlorophyll b and one derivate were detected. Pigments were analyzed separately and expressed as total carotenoids, total chlorophylls, and total pigments (Table 4).

As shown in Table 4, among the studied microalgae, the representatives of the Chlorophyta division showed the highest pigment content. The highest total pigment content of 508.51 mg/100 g of dry weight (DW) was determined in *Tetraselmis* sp. C6, followed by *Picochlorum* sp. D3 with 415.07 mg/100 g DW. These two microalgae strains also had the highest total chlorophyll (254.03 mg/100 g DW) and carotenoids content (298.89 mg/100 g DW), respectively. Almendinger et al. determined a high chlorophyll content in microalgae belonging to the Chlorophyta division, with the highest value of 60 mg/g determined in *N. oleobundans* [31]. Xanthophylls represented the most abundant carotenoid (50%) in all studied microalgae classes. *Picochlorum* sp. D3 had 233.39 mg of lutein per 100 g DW, which was the highest value among all studied microalgae in this research. For all three representatives of the Chlorophyta division, lutein was also the most abundant among carotenoids, followed by neoxanthin. A similar pigment profile with lutein as the most abundant (3.5 mg/g DW) was found in *Picochlorum* sp. HM1 [44]. Lutein is a highly valuable compound with immune-stimulating activity and a protective effect towards some inflammatory and chronic diseases. It is also an essential component of the eye macula, and due to free-radical scavenging ability protects the eye from oxidative stress [126]. Likewise, total carotenoids in *Tetraselmis* sp. C6 (254.48 mg/100 g DW) were comprised mostly of xanthophylls lutein and neoxanthin, with the highest content in all tested microalgae (79.44 mg/100 g DW). The content of lutein (164.84 mg/100 g DW) and neoxanthin was similar or even higher than those reported for the *Tetraselmis* strains [93,94,127,128]. Schüller et al. [128] conducted cultivation of *Tetraselmis* sp. CTP4 under comparable conditions to this study. Although a similar pigment profile was observed, the content of neoxanthin and lutein was markedly lower (0.5 mg neoxanthin/g DW and 1.64 mg lutein/g DW) compared to studied *Tetraselmis* sp. C6.

The pigment profile of *Tetraselmis* sp. Z3 was comparable to that of *Tetraselmis* sp. C6, but the content of each detected pigment was significantly lower. These differences could be attributed to specific strain characteristics [128,129]. The lowest content of each pigment as well as total pigments content was observed in the cyanobacterium *Euhalothece* sp. C1. It contained only 37.29 mg of total pigments per 100 g of DW, with lutein as the most abundant pigment (22.45 mg/100 g DW). In cyanobacteria, the main pigments are phycobiliproteins, while carotenoids have a protective role against saturating light and are a quencher of reactive oxygen species (ROS) [129]. Pigment phycocyanin is found as one of the main pigments in photosynthetic pigments of the cyanobacteria *Euhalothece* sp. C1, along with chlorophyll a, and carotenoids [57]. Microalgae belonging to the Bacillariophyta division had the highest content in fucoxanthin. Fucoxanthin is xanthophyll with strong antioxidative properties, found in diatoms, brown seaweed, and golden algae [130]. It has interesting medical and nutraceutical applications since it has health-promoting activities: antioxidant, anti-cancerogenic, anti-inflammatory, anti-obesity, anti-diabetic, neuroprotective and anti-angiogenic [19]. The fucoxanthin content in the studied diatoms was similar (40.11 mg/100 g DW and 39.54 mg/100 g DW in *Nitzschia* sp. S5 and *Nanofrustulum shiloi* D1, respectively). However, the observed fucoxanthin content was higher than reported for *Thalassiosira pseudonana* (0.2% DW), similar to that of *Thalassiosira* sp. (0.44 mg/g DW), but lower than that in *Skeletonema* sp. (1.81 mg/g DW) and *Chaetoceros* sp. (1.49 mg/g DW) [39,131]. Carotenoids comprised 73.56% and 72% of total pigments in *Nitzcshia* sp. S5, and *Picochlorum* sp. D3, respectively. These two microalgae showed the highest level of carotenoids among all studied strains.

### 2.6. Content of Total Phenol and Flavonoids and Antioxidant Activity

It is well known that carotenoids have a role as an antioxidant due to their quenching of reactive oxygen species generated during photosynthesis. Many studies have shown that carotenoids significantly contribute to the antioxidative capacity of microalgae [7,30,31,32,33,36]. However, the antioxidative capacity of microalgae cannot be attributed exclusively to carotenoids. Microalgal biomass contains different antioxidants, including aromatic compounds such as phenolic, flavonoids, polyunsaturated fatty acids, carotenoids, and other non-classified compounds [31,132]. The antioxidant activity was determined by ABTS and DPPH, using Trolox as a reference. The total phenolic content ranged from 5.99 ± 0.17 to 22.64 mg GAE/g DW between species, while total flavonoids varied from 0.14 to 0.67 mg QE/g DW (Table 5). The cyanobacterium *Euhalothece* sp. C1 presented the lowest values for both flavonoid and phenol content among all strains. The highest phenol content was measured for the *Tetraselmis* sp. C6 and *Nitzschia* sp. S5 extracts (22.33 and 22. 64 mg QE/g DW), and obtained values were similar to those reported in the literature [30,33,37,39,124]. The phenolic content of the *Tetraselmis* sp. C6 extract is comparable to the content reported in the work of Maadane et al. (25.5 mg GAE/g EW), and higher than found in the study of Goiris et al. (3.8 mg GAE/g DW) [32,133]. On the other hand, Haoujar et al. reported a bit higher content of 28.03 mg GAE/g for *Tetraselmis suecica*, and 39.34 mg GAE/g for diatom *Phaeodactylum tricornutum*, which is higher than the results obtained for two diatoms studied in this work. However, it should be mentioned that a comparison with the literature data is often difficult due to strain differences, but also the growth conditions (nutrients, temperature, stress application) that strongly influence the composition of the antioxidant compounds [133]. Furthermore, the methodology applied in analysis also affect the results, especially the solvent choice, as shown in several studies [35,37]. In addition, it is found that the content of phenolic substances in microalgae increases upon exposure to UV-light, that induces the antioxidative response to this type of stress [134,135]. The flavonoid content of the other investigated diatom, *Nanofrustulum* sp. D1, was similar *Picochlorum* sp. D3. However, *Picochlorum* sp. D3 had a higher content of total phenols [32,33,35,37]. To get a broader overview of the antioxidant profile of the investigated microalgae, two in vitro assays based on different mechanisms were conducted. The ABTS assay involves the initiation of peroxidation by generating a water-soluble peroxy radical to all known chain-breaking antioxidants, such as phenolic compounds and carotenoids [33]. The antioxidant activity measured, according to the TEAC assay, varied significantly between six strains from 12.75 to 170.96 µmol TE/g (Table 5). All microalgae extracts showed relatively high TEAC activity (40 µmol TE/g) except *Nanofrustulum shiloi* D1. Several authors reported significantly different antioxidant activities between the strains and species belonging to the same genus [7,32,37]. As expected, based on content of phenols, flavonoids and total carotenoids, the strain *Tetraselmis* sp. C6 showed the highest antioxidant activity using both assays.

The TEAC and DPPH scavenging activity of *Nitzschia* sp. S5 was much lower than in *Tetraselmis* sp. C6, even though the phenol and flavonoid contents were very much alike. It could be attributed to the higher carotenoid content in *Tetraselmis* sp. C6 (254.48 mg/100 g DW) compared to *Nitzschia* sp. S5 (101.2 mg/100 g DW). Diatom *Nitzschia* sp. S5 had higher antioxidant activity compared to two other representatives of the division Chlorophyte. In comparison to *Tetraselmis* sp. Z3, the diatom had higher levels of both phenols and carotenoids. Contrary, the content of the total pigments determined in *Picochlorum* sp. D3 was much higher than in *Nitzschia* sp. S5 (254.48 mg/100g and 101.2 mg/100g, respectively). However, *Nitzschia* sp. S5 had a higher phenol, and fucoxanthin content, exhibiting strong antioxidant activity, comprised a significant proportion of total carotenoids. Several studies showed that fucoxanthin has higher antioxidant activity than other carotenoids. Nomura et al. reported that fucoxanthin equimolarly reacted with DPPH as a radical quencher under anoxic conditions, while β-carotene, zeaxanthin, lycopene, lutein and β-cryptoxanthin scarcely reacted with DPPH and showed little or no quenching activities [136]. Fucoxanthin is an effective radical scavenger due to the allenic bonds, 5, 6-monoepoxide, two hydroxyl groups, a carbonyl group, and an acetyl group in the terminal ring, which are the major structural features that differed fucoxanthin from other carotenoids [19,137,138]. It has been suggested that the allenic bond was responsible for the higher antioxidant activity of fucoxanthin, in addition to six oxygen atoms and probably due to a higher sensitivity to radicals, especially under anoxic conditions [19]. A higher content of phenols and total carotenoids could explain the higher TEAC value for *Nitzschia* sp. S5 than the diatom *Nanofrustulum shiloi* D1 despite similar fucoxanthin content. Using the TEAC assay, Muller et al. demonstrated that the activity of carotenoids is determined by the number of conjugated double bonds, a hydroxyl group near the conjugated system and the substitution pattern of the β-ionone rings [38]. They also found that carotenoids are more active than xanthophylls in the TEAC assay than in FRAP assay [38]. For instance, the antioxidant activity of polyphenols is related to the degree and pattern of hydroxylation and the extent of conjugation in polyphenols [32].

The antioxidant activity of *Euhalothece* sp. C1 was similar to the activity of *Tetraselmis* sp. Z3 and *Nitzschia* sp. S5, although the cyanobacterium had the lowest content of phenols, flavonoids, and carotenoids. It is known that microalgae could produce a wide range of antioxidant compounds, including polysaccharides and long-chain polyunsaturated fatty acids, along with carotenoids and phenolic compounds. The antioxidant capacity of microalgae also depends on the solvent used for extraction. Antioxidant compounds in microalgal biomass have a different polarity, so extract composition strongly depends on the solubility of extracted compounds in the specific solvent [35,37,139]. The concentration of the sample that can scavenge 50% of DPPH free radicals (IC_50_) in the DPPH free radical scavenging method was also determined. The methanolic extract of *Tetraselmis* sp. C6 showed the lowest IC_50_ value (0.87 ± 0.23 mg mL^−1^). That value is comparable to 753.99 µg mL^−1^ reported for *Tetraselmis* sp. cultivated at low light [140], but higher than reported for *T. suecica* (394.40 µg mL^−1^) [30]. For all other investigated strains, the IC_50_ value was above 1 mg mL^−1^, with the maximum of 4.05 mg mL^−1^ for extract of *Nanofrustulum shiloi* D1. According to Coulombier et al., the IC_50_ values for *Nitzschia* sp. and *Tetraselmis* sp. were above 1000 µg mL^−1^ [140]. Some studies proved that the capacity of carotenoids to scavenge DPPH radical is weak, on average 50 times weaker than Trolox [38]. This observation agrees with the low capacity of a microalgae extract to quench ABTS and DPPH radicals, and have a much higher capacity to scavenge peroxyl radicals [140]. Further analysis of phenolic and flavonoid compounds is needed for elucidating possible synergistic, additive or antagonist interactions and their contribution to antioxidant activities.

### 2.7. Antimicrobial Activity of Microalgae Extracts

The antibacterial activity of microalgal methanolic extracts was tested against three Gram-negative bacteria (*Salmonella typhimurium, Escherichia coli*, and *Pseudomonas aeruginosa*), three Gram-positive bacteria (*Bacillus subtilis, Staphylococcus aureus*, and *Enterococcus faecalis*), the yeast *Candida utilis*, and the fungus *Aspergillus niger*. None of the tested microalgae showed antifungal activity against *A. niger*, and *C. utilis* (Table 6). Pure methanol was used as a negative control to determine its inhibitory effect on the growth of test microorganisms in the microalgal extracts. If the inhibition zone of the extract was larger than that of pure methanol, then the extract had antimicrobial activity. Antibiotic neomycin was used as a positive control for tested bacteria and nystatin for yeast and fungus. The inhibition activity of microalgal extracts on the growth of *C. utilis* can be attributed to the solvent effect since the inhibition zones were smaller than that of pure methanol. However, methanolic extracts showed antibacterial activity against all tested Gram-positive and Gram-negative bacteria. Previous findings also confirmed that ethanolic and methanolic extracts are highly effective against Gram-positive and Gram-negative bacteria [41]. All microalgal extracts, except *Tetraselmis* sp. Z3, inhibited the growth of *Escherichia coli*. The *Tetraselmis* sp. C6 extract had the most potent inhibitory effect on the growth of this bacterium. The extract of *Tetraselmis* sp. C6 had the strongest inhibition effect against *S. typhimurium* and *P. aeruginosa*, and *S. aureus* among all studied microalgae. Both *Tetraselmis* strains showed an inhibitory effect against *P. aeruginosa*. However, when comparing these two *Tetraselmis* strains, C6 showed stronger antimicrobial activity against all tested microorganisms compared to Z3 strain due to the lower extract concentration with the exception of E. faecalis which did not grow in presence of C6 extract. 

The inhibition zones measured with the other microalgae extract were lower than those obtained with pure methanol, indicating that inhibition was the result of the solvent effect. Diatoms *Nanofrustulum shiloi* D1 and *Nitzschia* sp. S5 showed strong inhibition against *B. subtilis*, with a 15 ± 1.41 and 20 ± 0.01 mm inhibition zone. Considering the concentration of the *N. shiloi* extract, the extract of *Nitzschia* sp. S5 showed higher antibacterial activity. These results are in accordance with results obtained with the extract of the diatom *Thalassiosira* sp. against *S. aureus* and *B. subtilis*. According to this study, the antibacterial activity of different diatoms depends on the strain, cultivation conditions and extraction method [34]. The methanolic extract of all studied microalgae strains showed an inhibitory effect on the growth of *S. aureus*. The highest inhibition effect was measured with the extract of *Tetraselmis* sp. C6 that had the lowest concentration among all tested microalgae. It has been earlier reported that the methanolic extracts of *Nostoc* spp., *Microcystis* spp., *Scenedesmus* spp., *Oscillatoria geminata*, and *Chlorella vulgaris* exerted a high antimicrobial potential against *S. aureus* and *B. subtilis*, with inhibition zone diameters between 16 and 18 mm [40]. In this study, the total inhibition of *E. faecalis* growth was obtained with the methanol extract of *Picochlorum* sp. D3. Cyanobacterium *Euhalothece* sp. C1 strongly inhibited the growth of *S. aureus* as well as *S. typhimurium*. Considering the results, methanolic extracts of microalgae investigated in this research showed good antimicrobial activity against bacterial strains, especially Gram-positive.

## 3. Materials and Methods

### 3.1. Microalgal Strains

The microalgae *Nitzschia* sp. S5, *Nanofrustulum shiloi* D1, *Picochlorum* sp. D3, *Tetraselmis* sp. Z3, *Tetraselmis* sp. C6 and cyanobacterium *Euhalothece* sp.C1 were isolated from the Adriatic Sea near Šibenik and Jadrija (latitude 43°43′40.01″ N; longitude 15°50′44.99″ E) and island Šolta (latitude 43°23′13″ N; longitude 16°17′15″ E) and identified by genetic barcoding analysis. DNA extraction was performed using NucleoSpin Tissue Kit (Macherey Nalgel; Hoerdt, Cedex, France). The 18S rRNA gene, the Internal Transcribed Spacer (ITS) of the rRNA operon and 28S rRNA gene were amplified by PCR from the genomic DNA [105]. PCR program was as follows: 5 min 95 °C; 30 s 94 °C, 30 s 56 °C, 1 min 72 °C and 10 min 72 °C for 35 cycles and then cooled at 4 °C. For purification Exo SAP-IT PCR Product Cleanup Reagent (ThermoFisher Scientific; Waltham, MA USA). For 18S rRNA gene following primers were used: SA (5′-AACCTGGTT-GATCCTGCCAGT-3′), SB (5′-TGATCCTTCTGCAGGTTCACCTAC-3′), 63F (5′-ATG-CTT-GTC-TCA-AAGATTA-3′) 1818R (5′-ACGGAAACCTTGTTACGA-3′), S69 (5′-ACCAGACTGCCCTCC- 3′) and S30 (5′-CGCGGTAATTGGAGCTCCA-3′). The ITS was amplified using primers 329F (5′-GTGAAC-CTG-CRG-AAG-GAT-CA-3′), D1R-R (5′- TATGCTTAAATTCAGCGGGT-3′) and ITS2F (5′-GGAGGCGCAGTAGCCAGCTGCCGT-3′). The 28S rRNA gene was amplified using primers D1R (5′-ACCCGCTGAATTTAAGCATA-3′) and D3Ca (5′-ACGAACGATTTGCACGTCAG-3′) [105]. The 16S rRNA gene fragments from cyanobacteria and plastids were amplified using the following primers: CYA106Fc (5′- CGGACGGGTGAGTAACGCGTGA-3′), CYA359Fc (5′-GGGGAATYT TCCGCAATGGG-3′), CYA781R (5′-GACTACTGGGGTATCTAATCCCATT-3′), and CYA781R (5′-GACTACAGGGGTATCTAATCCCTTT-3′) [103]. Amplified DNA fragments were sequenced and used for identification of the microalgae, which was performed through the NCBI BLAST. The results are presented in Supplementary Material. An overview of the studied species and their taxonomic classification are presented in Table 7. Five out of six microorganisms were identified to the genus level, and more detailed identification is necessary to identify them at a specie level.

Cultures are maintained in Guillard f/2 medium at 20 °C with artificial light provided by warm white light lamps with a luminous intensity of 3000 lux and 16:8 (h:h) light:dark photoperiod.

### 3.2. Microalgae Cultivation

All strains used were cultivated in Guillard f/2 medium. For the cultivation of microalgae belonging to Bacillariophyceae, sodium metasilicate was added. Chemicals used for growth media preparation were: NaNO_3_ (Kemika; Zagreb, Croatia), NaH_2_PO_4_ × H_2_O (Kemika; Zagreb, Croatia), Na_2_SiO_3_ × 9H_2_O (Sigma Aldrich; St. Louis, MO, USA), FeCl_3_ × 6H_2_O (Sigma Aldrich; St. Louis, MO, USA), Na_2_EDTA × 6H_2_O (Carlo Erba; Val-de-Reuil, France), MnCl_2_ × 4H_2_O (Kemika; Zagreb, Croatia), ZnSO_4_ × 7H_2_O (Kemika; Zagreb, Croatia), CoCl_2_ × 6H_2_O (Kemika; Zagreb, Croatia), CuSO_4_ × 5H_2_O (Kemika; Zagreb, Croatia), Na_2_MoO_4_ × 2H_2_O (Kemika; Zagreb, Croatia), thiamine (Acros Organics; Geel, Belgium), biotin (Sigma Aldrich; St. Louis, MO, USA) and cyanocobalamin (Sigma Aldrich; St. Louis, MO, USA).

The cultures for cultivation at a large-scale were prepared by weekly scale-up starting from 50 mL up to 2 L, using 10% (*v/v*) inoculum in f/2 medium, on rotary shaker 200 rpm and 23 °C.

Large-scale microalgae cultivation was conducted in 15 L containers with 8 L of medium and 1 L of mother culture. Biomass was harvested in the early stationary phase and subjected to biomass analysis. The culture was mixed by vigorous aeration with sterile humidified air at an airflow rate of 1.5 vvm. Cultures were grown under warm white light lamps with a luminous intensity of 3000 lux and 12:12 h light: dark photoperiod.

### 3.3. Characterization of Microalgal Strains

#### 3.3.1. Growth Kinetics

Microalgae growth was monitored by daily measurement of culture optical density at 540 nm (OD_540_) using Cary 100 UV-Vis spectrophotometer (Agilent; Santa Clara, CA, USA). Cell concentration was measured using the Thoma chamber and light microscope (OlympusCH20; Tokyo, Japan). At the end of cultivation, microalgae biomass was collected by centrifugation at 8000 rpm for 15 min at 4 °C (ThermoScientific, SL 8R Cell Culture Centrifuge; Waltham, MA, USA). Biomass was washed at least two times with distilled water to remove inorganic and organic compounds [141] and transferred in a dry and weighed glass tube. Biomass was dried by lyophilization and biomass concentration was calculated.

#### 3.3.2. Analysis of Biomass Composition

Lyophilized biomass was used for the analysis of biomass composition. All analyses were done in triplicates and results are presented as average value ± standard deviation.

##### Lipid Content and Fatty Acid Composition

Total lipids were determined as total fatty acid methyl esters (FAME) by in-situ transesterification applying protocol for analysis of microalgal biomass, developed by the National Renewable Energy Laboratory [142]. The FAME content was determined using Shimadzu GC-2010 Plus Capillary gas chromatograph (Shimadzu; Kyoto, Japan) equipped with a flame ionization detector (FID) and high-cyanopropyl capillary column (ZB-FAME 30 m × 0.25 mm × 0.2 µm; Phenomenex; Torrance, CA, USA). Samples were injected in split mode (split ratio 1:15) with AOC-20i injector. Helium was used as a carrier gas. The injector and detector temperatures were 250 and 260 °C, respectively. The temperature was programmed at 100 °C for 2 min, raised from 100 to 140 °C by a rate of 3 °C min^−1^, then from 140 °C to 190 °C by a rate of 3 °C min^−1^, then up to 260 °C at a rate of 30 °C min^−1^, and holding at that temperature for 2 min. Shimadzu LabStation Software was used for instrument control, data acquisition and data analysis (integration, retention times and peak areas). Identification of FAME was obtained by co-chromatography with commercially available FAME standard (Supelco FAME Mix, C4–C24; Catalog No.: 18919-1AMP, Bellefonte, PE, USA). Total lipids were determined as fatty acid methyl esters and calculated by the following equations (Equations (1) and (2)), where ODW stands for dry weight biomass used in the analysis (mg):

Total _FAME C13 normalized_ = Σ_C4–C24_(Amount_measured FAME Ci_/Amount_measured FAME C13_) × Amount_added C13_ (1)

% Total FAME = (Total_FAMEC13 normalized_/ODW _sample_) × 100 (2)

##### Lipid Classes Analysis

Analysis of lipid classes was done according to the procedure described in Gasparovic et al. [143]. Five milligrams of lyophilized samples was used to extract lipids using a modified one-phase solvent mixture of dichloromethane-methanol-water (Bligh and Dyer, 1959). In brief, 10 mL of one-phase solvent mixture dichloromethane-methanol-deionized water (1:2:0.8; *v:v:v*) and 15 μg of the internal standard were added and ultrasonicated for 3 min. Samples were stored overnight in the refrigerator. The next day, samples were filtered through a sinter funnel into a separatory funnel, additionally washed with 10 mL of the one-phase solvent mixture, then with 10 mL of two-phase solvent mixture of dichloromethane 0.73% NaCl solution (1:1; *v:v*), and finally with 10 mL of dichloromethane. Collected lipid extracts were dried under nitrogen and stored under −20 °C until analysis. Before analysis, samples were dissolved in 44 to 54 μL of dichloromethane.

Lipid classes were determined by thin-layer chromatography with flame ionization detection (TLC-FID, Iattroscan MK-Vi, Iatron, Japan) with a hydrogen flow of 160 mL min^−1^ and an airflow of 2000 mL min^−1^. Lipid classes were separated on silica-coated quartz TLC rods (Chromarods SIII) (SES-Analysesysteme, Bechenheim, Germany) and quantified by external calibration with a standard lipid mixture. For lipid classes separation, different solvent mixtures were used, followed by a subsequent partial burn of Chromarods. For 28 min, a mixture of hexane-diethyl ether-formic acid (97:3:0.2, *v:v:v*) was used to separate hydrocarbons, steryl esters and methyl esters. A mixture of hexane-diethyl ether-formic acid (80:20:0.2; *v:v:v*) was used for 30 min to separate triglycerides and free fatty acids, and the same solvent mixture was used for an additional 20 min to separate fatty alcohols, 1,3 diglycerides, sterols and 1,2 diglycerides, while a mixture of chloroform-acetone-formic acid (95:5:0.6; *v:v:v*) was used for 32 min to separate pigments and monoglycerides. For the separation of monogalactosyldiacylglycerol and digalactosyldiacylglycerol, acetone (100%) was used for 30 min. Sulfoquinovosyldiacylglycerol and phosphatidylglycerol were separated after 37 min in a mixture of acetone-chloroform-methanol-formic acid (33:33:33:0,6; *v:v:v:v*). The final mixture solvent, chloroform-methanol-ammonium hydroxide (50:50:5; *v:v:v*), was used for 35 min to separate phosphatidylethanolamine and phosphatidylcholine.

##### Carbohydrate Composition

Total carbohydrates in microalgae biomass were determined by a high-pressure liquid chromatography (HPLC) method developed by National Renewable Energy Laboratory [144]. After two-step sulfuric acid hydrolysis for degradation of polymeric forms of carbohydrates, monosaccharides were quantified by Ultra-performance liquid chromatograph (Agilent, 1290 Infinity II; Santa Clara, CA, USA), equipped with a refractive index detector (Agilent, Infinity II 1260; Santa Clara, CA, USA) and ion-exclusion HPLC column Rezex—Roa, Organic Acid H+ (8%) (Phenomenex; Torrance, CA, USA). The calibration curves of monosaccharides used as standards are presented in Table 8.

##### Total Protein Content

For total protein content determination, 1–1.3 mg of microalgal biomass was resuspended in 1 mL of 1 M NaOH (Merck, Darmstadt, Germany) and incubated in Thermo-shaker (BioSan TS-100; Riga, Latvia) for 20 min at 100 °C and 3000 rpm. The alkali biomass hydrolysate was cooled to room temperature and total proteins were determined according to the Lowry method [145].

##### Pigment Analysis

For extraction of pigments from lyophilized microalgae biomass, extraction conditions and procedure were carried out according to Repajić et al. [146]. High-performance liquid chromatography (HPLC) using UV-Vis photodiode-array (PDA) detection and data acquisition was used to analyze pigment extracts. The method for HPLC-UV-Vis/PDA pigments analysis was described previously [146].

##### Total Flavonoid Content

Total flavonoids were determined according to Bhattacharjya et al. (2020) [39]. Microalgal extract was prepared according to the method previously described in Section 3.4.1. A mixture of 0.2 mL of extract, 0.2 mL of 10% aluminium chloride, 0.2 mL of 1 M potassium acetate and 1.4 mL distilled water was prepared. The absorbance of the mixture was measured at 415 nm using Quercetin as standard.

##### Total Phenol Content

Determination of total phenol content was conducted according to the method described by Safafar et al. (2015) [33]. The mixture of 100 µL of microalgal extract (prepared by the procedure described in Section 3.4.1.), 600 µL of demineralized water, 500 µL of Folin–Ciocalteu reagent and 1500 µL of 20% (m v^−1^) sodium carbonate aqueous solution was prepared in the 10 mL volumetric flask. The flask was filled with water up to 10 mL. After incubation in the dark at room temperature for an hour, absorbance was measured at 750 nm. Gallic acid was used as standard, and the results were expressed as gallic acid equivalents per gram dry matter.

### 3.4. Antioxidant Scavenging Activity

#### 3.4.1. Preparation of Microalgal Extracts

Sixty milligrams of microalgae biomass were suspended in 600 µL of 100% methanol. The cell suspension was extracted at 50 °C in an ultrasound bath for one hour. The supernatant was collected in a clean glass vial by decanting and sealed. The extraction of biomass residue was repeated with another 600 µL of 100% methanol. Collected supernatants were combined and stored at −20 °C until analysis. The extract aliquot was evaporated to dryness and weighed. The extract concentration was calculated by dividing the extract weight by the volume and expressed in mg mL^−1^.

#### 3.4.2. The ABTS Radical Scavenging Assay

The antioxidant potential (radical scavenging capacity) of microalgal extracts was determined using ABTS assay according to Li et al. [37]. The stock solution of ABTS was prepared by mixing 88 µL of 140 mM potassium persulfate and 50 mL of 7 mM ABTS. All solutions used in the assay were prepared using 100% methanol. The mixture was left in the dark for 24 h. The working solution was prepared the next day by diluting the ABTS solution with methanol to give an absorbance at 734 nm (A_734_) of 0.7 ± 0.05. The 6-min reaction was started by adding 50 µL of extract to 1.9 mL of ABTS working solution and placing the mixture in the dark. Absorbance was measured at 734 nm, and total antioxidant activity was expressed as Trolox Equivalent (µmol TE g DW^−1^).

#### 3.4.3. The DPPH Free Radical Scavenging Assay

The DPPH assay was done as described in Assunção et al. (2017) [7]. The working solution was prepared by dissolving 4.8 mg of DPPH (Sigma, St. Louis, MO, USA) in 200 mL 100% methanol to a final concentration of 0.06 mM. Four concentrations of *Nitzschia* sp. S5 and *Tetraselmis* sp. C6 extracts and three different concentrations of *Tetraselmis* sp. Z3, *Picochlorum* sp. D3, *Nanofrustulum shiloi* D1 and *Euhalothece* sp. C1 extracts were prepared by 2, 4, 8 and 20-fold dilution with 100% methanol. The reaction was started by adding 200 µL to 1.8 mL of DPPH working solution. After a 15-min reaction in the dark, the absorbance was measured at 515 nm.

The antioxidant capacity of extracts was characterized by percent of inhibition (PI) and half-maximal inhibitory concentration (IC_50_) value:

PI = ((Abs_DPPH_ − Abs_sample_)/Abs_DPPH_) × 100 (3)

For the determination of IC_50_, the concentrations of extracts in mg mL^−1^ were plotted against the corresponding PI and calculated by linear regression (R^2^ ≥ 0.98) using the following equation:

IC_50_ = (50 − b)/m (mg mL^−1^) (4)

where b stands for Y-intercept, and m for slope of the linear equations.

#### 3.4.4. Preparation of Extracts for Antimicrobial Assay

Extracts were prepared by resuspending 15 mg of microalgal biomass in 150 µL of 100% methanol. Extraction was conducted at 25 °C in an ultrasound bath for one hour. The supernatant was collected in a clean glass vial and sealed. Collected supernatants were stored at −20 °C and used for the determination of antimicrobial potential. The extract aliquot was evaporated to dryness and weighed. The extract concentration was calculated by dividing the extract weight by the volume and expressed in mg mL^−1^.

#### 3.4.5. Disk Diffusion Antimicrobial Assay

Test microorganisms including bacteria *Bacillus subtilis*, *Staphylococcus aureus*, *Escherichia coli*, *Pseudomonas aeruginosa* and *Enterococcus faecalis*, fungus *Aspergillus niger* and yeast *Candida utilis* were cultivated in standard LB growth medium (10 g L^−1^ tryptone (Sigma Aldrich; St. Louis, MO, USA), 5 g L^−1^ yeast extract (Sigma Aldrich; St. Louis, MO, USA), 5 g L^−1^ sodium chloride (Nin Saltwork; Nin, Croatia), 20 g L^−1^ agar (Sigma Aldrich; St. Louis, MO, USA)). One hundred microliters of overnight culture were spread on solid LB agar in Petri dishes. Fifty microliters of microalgae extract were applied on a sterile disk and placed on the surface of a solid LB medium with a test microorganism. Neomycin and nystatin at 50 mg mL^−1^ were used as a positive control, while 100% methanol was used as a negative control. Plates with bacterial and fungal strains were incubated at 30 °C for 24 and 48 h, respectively. Afterwards, inhibition zones around the disk were measured. For all test microorganisms, assays were done in duplicate.

## 4. Conclusions

In this study, five strains of marine microalgae and one cyanobacterium were identified and characterized regarding macromolecular composition, pigment and fatty acid profiles, antioxidants, and antimicrobial activity. *Picochlorum* sp. D3 and *Nitzschia* sp. S5 (0.01 and 0.0092 h^−1^, respectively) had the highest growth rate, while the highest cell concentration was obtained by *Tetraselmis* sp. C6 and *Tetraselmis* sp. Z3 (0.45 and 0.48 g L^−1^). Microalgal biomass contained industrially interesting compounds for the production of food, feed, nutraceuticals and biofuels. The high lipid content obtained under unoptimized cultivation conditions makes *Nanofrustulum shiloi* D1 a potential feedstock for biodiesel production. *Tetraselmis* sp. Z3 had a high level of polyunsaturated fatty acids, including ω-3 fatty acid, eicosapentaenoic acid (EPA, C20:5), docosahexaenoic acid (DHA, C22:6) and alpha-linolenic (ALA, C18:3), and thus, it can be used as a fish oil replacement. All selected microalgae are a good source of proteins and pigments. Carotenoids were the most abundant pigments in all studied microalgae, with the highest content in strains of the Chlorophyta division. The methanolic extracts of all microalgal biomass showed moderate antioxidant activity. Furthermore, all microalgal extracts strongly inhibited the growth of Gram-negative *E. coli* and *S. typhimurium* and Gram-positive *S. aureus.*

## Figures and Tables

**Table 1:** Biomass concentration (X), biomass productivity (Pr) and specific growth rate (μ).

Strain	X (g L^−1^)	Pr_x_ (mg L^−1^ day^−1^)	µ (day^−1^) *
*Nitzschia* sp. S5	0.28 ± 0.14	19.74 ± 3.25	0.15 ± 0.003
*Nanofrustulum shiloi* D1	0.10 ± 0.01	12.8 ± 3.78	0.099 ± 0.006
*Picochlorum* sp. D3	0.41 ± 0.01	33.98 ± 0.02	0.217 ± 0.089
*Tetraselmis* sp. Z3	0.48 ± 0,11	28.00 ± 0.11	0.064 ± 0.022
*Tetraselmis* sp. C6	0.45 ± 0.04	26.29 ± 2.14	0.076 ± 0.027
*Euhalothece* sp. C1	0.28 ± 0.08	24.42 ± 1.64	0.095 ± 0.002

* lnX = µ·t + lnX_0_; Exponential growth: *Nitzschia* sp. S5 (4–12th day); *Nanofrustulum shiloi* D1 (2–12th day); *Picochlorum* sp. D3 (2–7th day); *Tetraselmis* sp. Z3 (6–13th day); *Tetraselmis* sp. C6 (6–14th day); *Euhalothece* sp. C1 (2–10th day).

**Table 2:** Fatty acid composition of microalgal lipids.

	Fatty Acids (%, g g^−1^)					
	*Nitzschia* sp. S5	*Nanofrustulum* *shiloi* D1	*Picochlorum* sp. D3	*Tetraselmis* sp. Z3	*Tetraselmis* sp. C6	*Euhalothece* sp. C1
11:0	—	—	—	—	—	1.45 ± 0.15
14:0	10.59 ± 0.32	0.66 ± 0.34	0.38 ± 0.08	—	—	—
14:1 cis 9	-	0.07 ± 0.01	—	—	—	—
15:0	—	0.19 ± 0.14	—	—	—	—
15:1 cis 10	—	2.45 ± 2.98	—	—	—	—
16:0	23.19 ± 0.26	30.76 ± 0.27	28.14 ± 2.61	31.63 ± 1.1	33.25 ± 1.35	28.40 ± 0.45
16:1 cis 9	56.12 ± 0.14	41.57 ± 2.18	1.36 ± 0.03	3.54 ± 0.12	2.24 ± 0.09	14.07 ± 0.03
17:0	1.06 ± 0.002	0.57 ± 0.14	22.23 ± 2.47	1.02 ± 0.23	2.62 ± 0.03	48.79 ± 0.42
17:1 cis 10	4.75 ± 0.15	1.88 ± 0.73		0.77 ± 0.31	2.90 ± 0.18	—
18:1 cis 9	—	0.67 ± 0.17	2.92 ± 0.65	16.56 ± 0.63	9.55 ± 0.37	0.84 ± 0.26
18:2 trans 9, 12	—	—	—	—	—	2.54 ± 0.31
18:2 cis 9, 12	—	1.88 ± 0.55	33.33 ± 1.76	8.5 ± 0.13	12.69 ± 0.39	3.99 ± 0.7
18:3 cis 9, 12, 15	—	0.72 ± 0.34	11.59 ± 0.91	22.50 ± 0.47	21.75 ± 0.82	—
20:1 cis 11	—	0.14 ± 0.03	—	3.81 ± 0.45	—	—
20:3 cis 8, 11, 14	—	2.41 ± 3.73	—	—	—	—
20:4 cis 5, 8, 11, 14	—	9.29 ± 3.3	—	—	1.32 ± 0.03	0.18 ± 0.04
22:1 cis 13	—	—	—	—	—	—
20:5 cis 5, 8, 11, 14, 17	3.48 ± 0.19	6.79 ± 1.84	—	8.58 ± 1.05	7.01 ± 3.17	—
24:1 cis 15	—	0.19 ± 0.05	—	—	—	—
22:6 cis 4, 7, 10, 13, 16, 19	—	0.47 ± 0.37	—	1.97 ± 0.78	—	—
SFA	34.84 ± 0.06	32.35 ± 0.85	52.98 ± 2.33	33.81 ± 1.68	36.88 ± 1.38	78.65 ± 0.93
MUFA	64.68 ± 0.13	46.08 ± 5.40	7.74 ± 3.35	24.66 ± 0.58	18.19 ± 0.61	14.70 ± 0.61
PUFA	3.48 ± 0.19	21.57 ± 6.09	39.28 ± 3.95	41.53 ± 1.11	44.93 ± 1.99	6.65 ± 0.84

**Table 3:** Lipid classes composition.

	Lipid Class (%, g g^−1^)					
	*Nitzschia* sp. S5	*Nanofrustulum* *shiloi* D1	*Picochlorum* sp. D3	*Tetraselmis* sp. Z3	*Tetraselmis* sp. C6	*Euhalothece* sp. C1
Neutral lipids	46.60	68.36	17.93	17.07	15.22	18.58
monoglicerides	6.24	1.12	2.95	0.00	1.45	0.00
1,2 diglycerides	0.00	0.45	0.24	0.31	0.59	0.15
1,3 diglycerides	0.00	0.00	0.96	0.00	0.31	0.00
triglycerides	0.00	0.71	5.12	0.59	0.58	0.90
free fatty acids	37.35	58.34	5.80	10.25	6.86	15.23
sterols	1.30	2.03	2.53	2.21	2.77	0.61
steryl esters	1.71	5.71	0.34	3.71	2.66	1.69
Polar lipids	49.33	29.03	78.33	77.25	76.29	74.59
Glycolipids	19.08	13.30	33.97	21.77	26.31	17.02
monogalactosyldiacylglycerol	8.46	8.71	22.48	7.19	9.29	4.66
digalactosyldiacylglycerol	1.67	0.58	1.69	1.35	2.14	0.59
sulfoquinovosyldiacylglycerol	8.95	4.02	9.80	13.23	14.89	11.78
Phospholipids	30.25	15.74	44.36	55.47	49.98	57.56
phosphatidylglycerol	21.74	10.00	21.93	36.32	36.08	36.83
phosphatidylethanolamine	8.24	5.25	14.35	18.67	13.26	19.88
phosphatidylcholine	0.27	0.50	8.08	0.49	0.63	0.85
hydrocarbon	1.94	1.53	2.77	2.10	3.41	3.77
pigments	2.13	1.06	0.96	3.58	5.09	3.07

**Table 4:** Pigment composition of isolated marine microalgae strains.

	Pigment (mg/100 g DW)					
	*Nitzschia* sp. S5	*Nanofrustulum* *shiloi* D1	*Picochlorum* sp. D3	*Tetraselmis* sp. Z3	*Tetraselmis* sp. C6	*Euhalothece* sp. C1
Fucoxanthin	40.11	39.54	2.93	4.91	7.59	—
Neoxanthin	5.53	—	14.92	11.62	79.44	2.19
Lutein	55.56	—	233.39	37.12	164.84	22.45
Canthaxanthin	—	—	47.54	—	—	—
α-carotene	—	—	—	—	1.6	—
β-carotene	—	0.36	0.11	0.19	1.01	0.13
Chlorophyll b	—	—	—	19.85	97.91	—
Chlorophyll a	36.38	131.07	116.18	31.5	156.12	12.52
Total pigments	137.58	170.97	415.07	105.19	508.51	37.29

**Table 5:** Total flavonoid, phenol and carotenoid content and total antioxidative properties of microalgal extracts.

Extract * of	*Nitzschia* sp. S5	*Nanofrustulum* *shiloi* D1	*Picochlorum* sp. D3	*Tetraselmis* sp. Z3	*Tetraselmis* sp. C6	*Euhalothece* sp. C1
Total flavonoid (mg quercetin/g DW)	0.67 ± 0.10	0.38 ± 0.01	0.37 ± 0.02	0.21 ± 0.05	0.44 ± 0.02	0.14 ± 0.02
Total phenols (mg GAE/g DW)	22.64 ± 1.86	11.67 ± 0.27	16.53 ± 2.87	6.51 ± 0.28	22.33 ± 0.24	5.99 ± 0.17
Total carotenoids (mg/100 g DW)	101.2	39.9	298.89	53.84	254.48	24.77
ABTS (µmol TE/g)	75.82 ± 7.88	12.75 ± 4.65	54.42 ± 0.04	51.36 ± 5.11	170.96 ± 5.26	73.83 ± 1.40
DPPH (µmol TE/g)	86.93 ± 3.27	36.53 ± 0.59	19.51 ± 1.36	80.27 ± 1.86	199.97 ± 5.51	90.69 ± 7.43
IC_50_ (mg/mL)	1.82 ± 0.08	4.05 ± 0.19	1.90 ± 0.12	2.05 ± 0.26	0.87 ± 0.23	1.80 ± 0.16

* concentration of extracts (mg/mL): *Nitzschia* sp. S5 (5.20 ± 0.03); *Nanofrustulum shiloi* D1 (10.76 ± 0.08); *Picochlorum* sp. D3 (10.49 ± 0.05); *Tetraselmis* sp. Z3 (4.79 ± 0.57); *Tetraselmis* sp. C6 (2.92 ± 0.38); *Euhalothece* sp. C1 (5.91 ± 0.04).

**Table 6:** Antibacterial activity of microalgae extracts tested by disk diffusion method.

Extract * of	Inhibition Zone (mm ± s.d.)						K^+^ (neomycin ^1^/nystatin ^2^)	K^−^ (methanol)
	*Nitzschia* sp. S5	*Nanofrustulum* *shiloi* D1	*Picochlorum* sp. D3	*Tetraselmis* sp. Z3	*Tetraselmis* sp. C6	*Euhalothece* sp. C1		
*E. coli*	11.00 ± 0.01	12.00 ± 0.01	13.00 ± 0.01	—	26.05 ± 0.07	13.10 ± 0.14	—	—
*S. typhimurium*	10.50 ± 0.71	9.05 ± 0.07	9.00 ± 0.09	—	20.00 ± 4.24	17.50 ± 4.95	27 ± 2.12 ^1^	—
*P. aeruginosa*	9.50 ± 3.54	9.50 ± 2.12	9.00 ± 0.07	16.70 ± 0.42	17.00 ± 0.09	12.00 ± 1.41	41 ± 3.0 ^1^	15 ± 1.41
*B. subtilis*	15.00 ± 1.41	20.00 ± 0.01	8.50 ± 0.71	—	10.00 ± 0.07	—	21 ± 2.0 ^1^	9 ± 0.71
*S. aureus*	9.05 ± 0.07	10.00 ± 0.01	14.50 ± 2.12	14.50 ± 0.71	22.00 ± 0.1	26.00 ± 1.26	21 ± 1.5 ^1^	—
*E. faecalis*	—	—	no growth	18.00 ± 4.24	—	9.00 ± 0.19	19 ± 2.0 ^1^	11 ± 0.58
*C. utilis*	11.00 ± 4.24	9.00 ± 0.07	9.00 ± 0.14	7.05 ± 0.07	12.00 ± 4.24	5.50 ± 2.12	21 ± 1.7 ^2^	13 ± 2.12
*A. niger*	—	—	—	—	—	—	30 ± 1.45 ^2^	—

* concentration of extracts (mg mL^−1^): *Nitzschia* sp. S5 (10.63 ± 0.34); *Nanofrustulum shiloi* D1 (22.10 ± 0.52); *Picochlorum* sp. D3 (21.76 ± 0.52); *Tetraselmis* sp. Z3 (10.72 ± 0.15); *Tetraselmis* sp. C6 (6.39 ± 0.01); *Euhalothece* sp. C1 (12.18 ± 0.39); ^1^ neomycin was used as a positive control for bacteria cultures and ^2^ nystatin for yeast *C. utilis* and fungus *A. niger.*

**Table 7:** Overview of isolated microalgae species and their taxonomy.

Division	Isolate Name	Closest Named Species	Origin (Location)	Identity
Bacillariophyta	*Nitzschia* sp. S5	*Nitzschia* sp.	Jadrija; Croatia	97.90%
Bacillariophyta	*Nanofrustulum shiloi* D1	*Nanofrustulum shiloi*	Island Šolta, Croatia	99.27%
Chlorophyta	*Picochlorum* sp. D3	*Picochlorum* sp.	Šibenik; Croatia	99.87%
Chlorophyta	*Tetraselmis* sp. Z3	*Tetraselmis rubens* or *Tetraselmis marina*	Jadrija; Croatia	100% 100%
Chlorophyta	*Tetraselmis* sp. C6	*Tetraselmis suecica* or *Tetraselmis rubens*	Jadrija; Croatia	96.46% 96.46%
Cyanobacteria	*Euhalothece* sp. C1	*Euhalothece* sp. or *Halothece* sp.	Island Šolta, Croatia	97.94% 97.06%

**Table 8:** Calibration curves of monosaccharide standards analyzed by UPLC.

Monosaccharide (g L^−1^)	Calibration Curve Equation	Determination Coefficient
glucuronic acid	y = 113192x + 44.361	0.99
glucose	y = 135278x − 3377	0.99
mannose	y = 128302x − 5039.7	0.99
galactose	y = 132077x + 987.09	0.99
xylose	y = 129878x − 627.53	0.99
fructose	y = 125211x + 3745.8	1.00
rhamnose	y = 113900x + 479.24	0.99
arabinose	y = 128443x − 3314.1	0.99
fucose	y = 136441x − 2965.8	0.99
glucosamine hydrochloride	y = 128.54x + 279.1	0.98

## Data Availability

The data presented in this study are available for a limited time, on request from the corresponding author.

## Supplementary Materials

The following are available online, Figure S1: Microscopic pictures of isolate D1, Figure S2: Isolate D1 (a) sequence of DNA fragment of 18S rRNA gene; (b) BLAST results, Figure S3: Isolate D1 (a) sequence of DNA fragment of ITS gene; (b) BLAST results, Figure S4: Isolate D1 (a) sequence of DNA fragment of LSU gene; (b) BLAST results, Figure S5: Microscopic pictures of isolate Z3, Figure S6: Isolate Z3 (a) sequence of DNA fragment of 18S rRNA gene; (b) BLAST results, Figure S7: Microscopic pictures of isolate D3, Figure S8: Isolate D3 (a) sequence of DNA fragment of 18S rRNA gene; (b) BLAST results, Figure S9: Isolate D3 (a) sequence of DNA fragment of 16S rRNA gene; (b) BLAST results, Figure S10: Isolate D3 (a) sequence of DNA fragment of LSU gene; (b) BLAST results, Figure S11: Microscopic pictures of isolate C6, Figure S12: Isolate C6 (a) sequence of DNA fragment of 18S rRNA gene; (b) BLAST results, Figure S13: Isolate C6 (a) sequence of DNA fragment of LSU gene; (b) BLAST results, Figure S14: Microscopic pictures of isolate S5, Figure S15: Isolate S5 (a) sequence of DNA fragment of 18S rRNA gene; (b) BLAST results, Figure S16: Microscopic pictures of isolate C1, Figure S17: Isolate C1 (a) sequence of DNA fragment of 16S rRNA gene; (b) BLAST results, Table S1: Identification of isolate D1, Table S2: Identification of isolate Z3, Table S3: Identification of isolate D3, Table S4: Identification of isolate C6, Table S5: Identification of isolate S5, Table S6: Identification of isolate C1.
