# Supplementary material for: Bioprospecting of Microalgae Isolated from the Adriatic Sea: Characterization of Biomass, Pigment, Lipid and Fatty Acid Composition, and Antioxidant and Antimicrobial Activity
Source: Molecules. 2022 Feb 12;27(4):1248. doi: 10.3390/molecules27041248 (PMC8875609; doi:10.3390/molecules27041248)
Supplement: Supplementary file 1 [file molecules-27-01248-s001.zip › molecules-1527988-supplementary.pdf]

Supplementary Material

Figure S1. Microscopic pictures of isolate D1

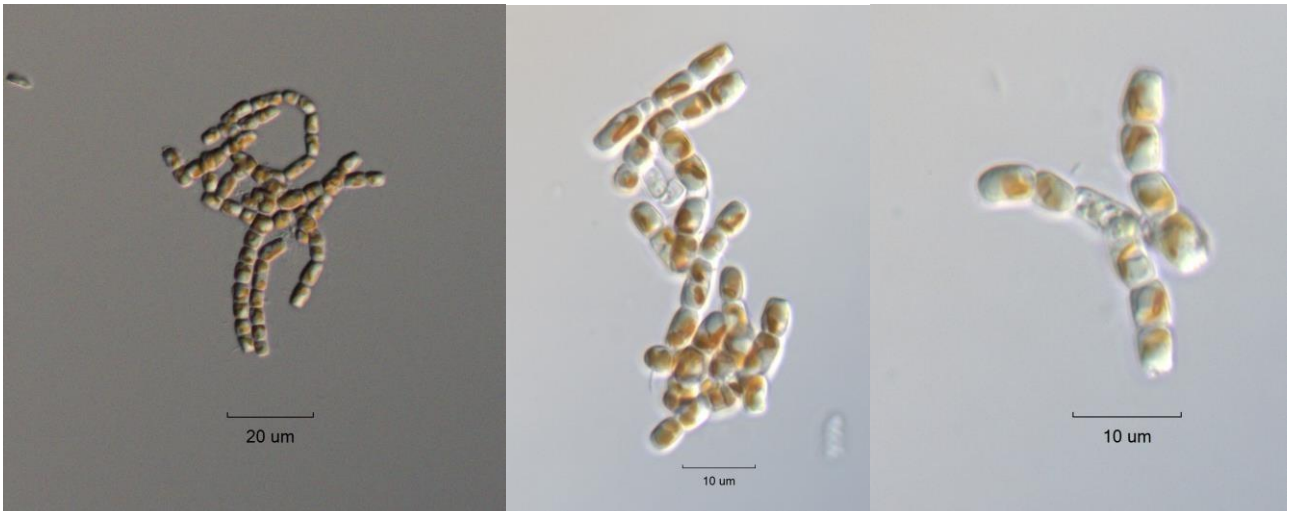

Figure S2. Isolate D1 a) sequence of DNA fragment of 18S rRNA gene; b) BLAST results (NCBI database http://www.ncbi.nlm.nih.gov/)

a)

>Gene 18 rRNA:
GCTCGTAGTTGGATTTGTGGGTGGAGCGAGCGTCCCGACGCTCAGCGTGGGTGAGGTTCGTCTCCGTCCATCCTTGGGTGGAATCCGCGTGGCATTGAGTTGTCGTGCGGGGGATGC
CCATCTTTTACTGTGAAAAAATTAGAGTGTTCAAAGCAGGCTTACGCCGTTGAATATATTAGCATGGAATAATGAGATAGGACCTGGGTACTATTTTGTTGGTTTGCGCACCGAGGTAA
TGATTAATAGGGACAGTTGGGGGTATTCGTATTCCATTGTCAGAGGTGAAATTCTTGGATTTCTGGAAGACGAACGACTGCGAAAGCATTTACCAAGGATGTTTTCATTAATCAAGAA
CGAAAGTTAGGGGATCGAAGATGATTAGATACCATCGTAGTCTTAACCATAAACTATGCCGACAAGGGATTGGTGGGCGTTTGTATGACCCCATCAGCACCTTAGGAGAAATCACAA
GTTTTTGGGTTCCGGGGGGAGTATGGTCGCAAGGCTGAAACTTAAAGAAATTGACGGAAGGGCACCACCAGGAGTGGAACCTGCGGCTTAATTTGACTCAACACGGGAAAACTTAC
CAGGTCCAGACACAGTGAGGATTGACAGATTGAGAGCTCTTTCTTGATTCTGTGGGTGGTGGTGCATGGCCGTTCTTAGTTGGTGGAGTGATTTGTCTGGTTAATTCCGTTAACGAAC
GAGACCCCTGCCTGCTAAATAGTTGGGCGAATGAATGTTCATTGGCTGCAGCTTCTTAGAGGGACGTGCGTTCTATTAGACGCAGGAAGATAGGGGCAATAACAGGTCTGTGATGCC
CTTAGATGTCCTGGGCCGCACGCGCGTTACACTGATGCACTCAACGAGTCTACCTTGGCCGAGAGGCCTGGGAAATCTTGTTAACATGCATCGTGATAGGGATAGATTATTGCAATTA
TTAATCTTGAACGAGGAATTCCTAGTAAACGCAGATCATCAATCTGCATTGATTACGTCCCTGCCCTTTGTACACACCGCCCGTCGCACCTACCGATTGAATGGTCCGGTGAAGCCTCG
GGATTGCGATGAGTTTCCTTCATTGGGAGTTTGTCGCGAGAACTTGTCTAAACCT

b)

Description	Scientific Name	Max Score	Total Score	Query Cover	E value	Per. Ident	Acc. Len	Accession
Nanofrustulum shiloi isolate CCMP1306 18S ribosomal RNA gene, partial sequence	Nanofrustulum …	2052	2052	100%	0.0	99.91%	1203	MF093126.1
Nanofrustulum shiloi strain CCMP1306 18S ribosomal RNA gene, partial sequence	Nanofrustulum …	2050	2050	100%	0.0	99.82%	1904	AY485505.1
Nanofrustulum cf. shiloi strain TAU-MAC 2215 small subunit ribosomal RNA gene, partial sequence	Nanofrustulum …	2047	2047	100%	0.0	99.82%	1204	MN510855.1
Nanofrustulum shiloi small subunit ribosomal RNA gene, partial sequence	Nanofrustulum …	2047	2047	100%	0.0	99.82%	1741	AF525658.1
Fragilaria pinnata 18S ribosomal RNA gene, partial sequence	Fragilaria pinnata	2041	2041	100%	0.0	99.73%	1211	EF192988.1
Nanofrustulum shiloi clone P194 small subunit ribosomal RNA gene, partial sequence	Nanofrustulum …	2037	2037	100%	0.0	99.64%	1786	EF491891.1
Nanofrustulum shiloi partial 18S rRNA gene, strain p194	Nanofrustulum …	2025	2025	100%	0.0	99.46%	1783	AM746971.1
Nanofrustulum cf. shiloi strain TAU-MAC 2115 small subunit ribosomal RNA gene, partial sequence	Nanofrustulum …	2023	2023	99%	0.0	99.64%	1174	MN510854.1
Nanofrustulum wachnickianum isolate SZCZCH194 18S ribosomal RNA gene, partial sequence	Nanofrustulum …	2002	2002	100%	0.0	99.10%	1732	KU851874.1
Pseudostaurosira elliptica isolate s0398 18S ribosomal RNA gene, partial sequence	Pseudostauros…	1991	1991	100%	0.0	98.92%	1732	MF093080.1
Pseudostaurosira brevistriata gene for 18S rRNA, partial sequence, strain: s0398	Pseudostauros…	1991	1991	100%	0.0	98.92%	1779	AB430608.1
Staurosira sp. I-141 18S ribosomal RNA gene, partial sequence	Staurosira sp. I…	1991	1991	100%	0.0	98.92%	1791	EF465491.1
Pseudostaurosira elliptica isolate SZCZCH1348 18S ribosomal RNA gene, partial sequence	Pseudostauros…	1986	1986	100%	0.0	98.83%	1781	KU851878.1
Pseudostaurosira elliptica isolate SZCZCH1249 18S ribosomal RNA gene, partial sequence	Pseudostauros…	1980	1980	100%	0.0	98.74%	1198	KU851872.1

Figure S3. Isolate D1 a) sequence of DNA fragment of ITS gene; b) BLAST results (NCBI database http://www.ncbi.nlm.nih.gov/)

a)

Gene ITS:
TCATTGAAACTTTGAACGCACATTGCGCTTCCGGGCCATTCCCGGTAGCATACTTATCTGAGTGTCCGCGAACCCCACTCAGCGTGTGTGCCATCAATCAATAGTGGCGCGTAC
TGCTGGACTGTGGCTCTACAACAATTTATTCTTGTTGAGTCCAAGTGAGATGACAACCTCCTTGCGCATTTGACCCCTTCCAATGAGGGGTGTCTGAGCTAGTTTGTTCCATCGT
GGAATCGAACGGGGATGTTCCTAGTAGGGCGGAGCCAGCCTCCATCTAATCCAGGAACTCGTCTTCTGCCGAGACCATACCATTGAATAAGCCAGCCCAA

b)

Description	Scientific Name	Max Score	Total Score	Query Cover	E value	Per. Ident	Acc. Len	Accession
Fragilaria pinnata culture-collection CCMP:398 5.8S ribosomal RNA gene and internal transcribed sp...	Fragilaria pinn...	126	126	26%	1e-24	93.02%	419	GQ330340.1
Tabularia cf. tabulata strain CCMP846 5.8S ribosomal RNA gene and internal transcribed spacer 2, p...	Tabularia cf. ta...	124	124	23%	4e-24	96.05%	406	EU883364.1
Grammatophora sp. B LG-2014 5.8S ribosomal RNA gene, partial sequence; internal transcribed spa...	Grammatopho...	122	122	25%	2e-23	92.86%	609	KJ671768.1
Tabularia affinis strain 206AUK 5.8S ribosomal RNA gene and internal transcribed spacer 2, partial s...	Tabularia affinis	122	122	25%	2e-23	92.86%	347	EU883366.1
Fragilaria sp. RCC4616 internal transcribed spacer 1, partial sequence; 5.8S ribosomal RNA gene, c...	Fragilaria sp. ...	121	121	26%	6e-23	91.86%	923	MH843527.1
Grammonema striatula culture-collection CCMP:1094 5.8S ribosomal RNA gene and internal transcri...	Grammonema...	121	121	26%	6e-23	91.86%	426	GQ330341.1
Grammonema striatula voucher IK_MTA4 5.8S ribosomal RNA gene, partial sequence	Grammonema...	121	121	26%	6e-23	91.86%	435	FJ864286.1
Grammatophora oceanica voucher IK_MTA110 5.8S ribosomal RNA gene, partial sequence	Grammatopho...	119	119	26%	2e-22	90.70%	424	FJ864287.1

Figure S4. Isolate D1 a) sequence of DNA fragment of LSU gene; b) BLAST results (NCBI database http://www.ncbi.nlm.nih.gov/)

a)

Gene LSU:
CATATAATTAAGCGGAGGAAAAGAAACTAACCAGGATTCCCCTAGTAAGGGCGACTGAAGCGGGAAGAGCCCAACATGCGAATCTGTGCCTTGGCACCGAATTGTGGTCTGTTCG
CGCTACGTGGCTCGGGCCAAGTCCCTTGGAAAAGGGCAGCTGAGAGGGTGAGACTCCCGTCCGCCCGAGCCAGTGAGCGAGCATGTTGGAGTCGAGTTGTTTGGGATTGCAGCT
CAAAGCGGGTGGTAAATTCCATCGAAGGCTAAATATTGGTGGGAGACCGATAGCGTACAAGTACCGTGAGGGAAAGATGCAAAGAACTTTGAAAAGAGAGTTAAAGAGTACCTG
AAATTGCTGAAAGGGAAGCGAAGGAAACCAGTGTTGCTGCGCAGTCATACTTCTCCGTCCGCTTGCGGGCGGGGCGCTGTGTCTGCGCTCGGGTCAGGCTTGGTTGGGGGCGCC
GCAAAGGCTCCTCTGGGAGTTGACCGCTGGAGCTGGCGGTGGTCCCGACCGAGGTTGGCGAAATGGTTTTCTTTACCCCGTCTTGAAA

b)

Description	Scientific Name	Max Score	Total Score	Query Cover	E value	Per. Ident	Acc. Len	Accession
Nanofrustulum shiloi gene for 28S rRNA, partial sequence, strain: p194	Nanofrustulum shiloi	989	989	100%	0.0	99.27%	574	AB430640.1
Pseudostaurosira brevistriata gene for 28S rRNA, partial sequence, strain: s0398	Pseudostaurosira brevistriata	837	837	100%	0.0	94.32%	572	AB430648.1
Cyclotella caspia 28S ribosomal RNA gene, partial sequence	Cyclotella caspia	754	754	98%	0.0	91.91%	549	AY496197.1
Opephora sp. s0357 gene for 28S rRNA, partial sequence, strain: s0357	Opephora sp. s0357	734	734	100%	0.0	90.94%	572	AB430643.1
Plagiostriata goreensis gene for 28S rRNA, partial sequence, strain: s0388	Plagiostriata goreensis	645	645	100%	1e-180	87.96%	572	AB430644.1
Asterionella formosa gene for 28S rRNA, partial sequence, strain: s0339	Asterionella formosa	628	628	100%	1e-175	87.73%	563	AB430632.1
Diatoma moniliforme gene for 28S rRNA, partial sequence, strain: s0383	Diatoma moniliforme	616	616	100%	1e-171	87.36%	563	AB430635.1
Eunotia bilunaris voucher SPAG24 28S ribosomal RNA gene, partial sequence	Eunotia bilunaris	614	614	100%	4e-171	87.39%	892	MH272973.1
Eunotia bilunaris voucher NOS14 28S ribosomal RNA gene, partial sequence	Eunotia bilunaris	614	614	100%	4e-171	87.39%	879	MH272969.1

Table S1. Identification of isolate D1

**Identification of isolate D1**	
Domain	Eukaryota
Division	Bacillariophyta
Class	Bacillariophyceae
Order	Fragilariales
Famille	Staurosiraceae
Genus	*Nanofrustulum*
Species	*shiloi*

Figure S5. Microscopic pictures of isolate Z3

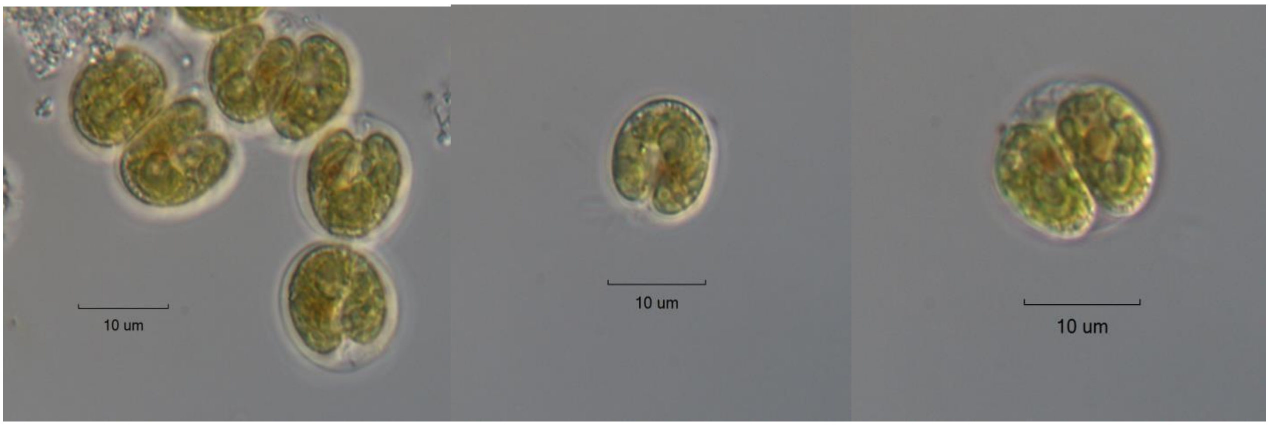

Figure S6. Isolate Z3 a) sequence of DNA fragment of 18S rRNA gene; b) BLAST results (NCBI database http://www.ncbi.nlm.nih.gov/)

a)

Gene: 18S rRNA:

CTGCGAATGGCTCATTAAATCAGTTATAGTTTATTTGATGGTACCTACTACTCGGATAACCGTAGTAATTCTAGAGCTAATACGTGCGTAAATCCCGACTTCTGGAAGGGACGTATTTATT
AGATTTAAGGCCAACCGAGCTCTGCTCGTCTCTTGGTGAATCATGATAACTTCACGAATCGCATGGCCTCCGCGCCGGCGATGTTTCATTCAAATTTCTGCCCTATCAATTGGCGATGGTA
GGATAGAGGCCTACCATGGTGTTAACGGGTGACGGAGGATTAGGGTTCGATTCCGGAGAGGGAGCCTGAGAAATGGCTACCACATCCAAGGAAGGCAGCAGGCGCGCAAATTACCCA
ATCCTGATACAGGGAGGTAGTGACAATAAATAACAATACCGGGCTTTTCAAGTCTGGTAATTGGAATGAGTACAATCTAAATCCCTTAACGAGGATCCATTGGAGGGCAAGTCTGGTGC
CAGCAGCCGCGGTAATTCCAGCTCCAATAGCGTATATTTAAGTTGTTGCAGTTAAAAAGCTCGTAGTTGGATTTCGGATGGGACTTGCCGGTCCGTCGTTGCGATGTGCACTGGCCAGTC
CTATCTTGTTGTCGGGGACTAGCTCCTGGGCTTCACTGTCCGGGACTAGGAGCTGACGAGGTTACTTTGAGTAAATTAGAGTGTTCAAAGCAAGCCTACGCTCTGAATACATTAGCATGG
AATAACACGATAGGACTCTGGCTTATCCTGTTGGTCTGTGAGACCAGAGTAATGATTAAGAGGGACAGTCGGGGACATTCGTATTTCATTGTCAGAGGTGAAATTCTTGGATTTATGAA
AGACGAACTTCTGCGAAAGCATTTGTCAAGGATGTTTTCATTAATCAAGAACGAAAGTTGGGGGCTCGAAGACGATTAGATACCGTCCTAGTCTCAACCATAAACGATGCCGACTAGGG
ATTGGCAGACGTTTTTTTGATGACTCTGCCAGCACCTTATGAGAAATCAAAGTTTTTGGGTTCCGGGGGGAGTATGGTCGCAAGGCTGAAACTTAAAGGAATTGACGGAAGGGCACCAC
CAGGCGTGGAGCCTGCGGCTTAATTTGACTCAACACGGGAAAACTTACCAGGTCCAGACATAGTGAGGATTGACAGATTGAGAGCTCTTTCTTGATTCTATGGGTGGTGGTGCATGGCC
GTTCTTAGTTGGTGGGTTGCCTTGTCAGGTTGATTCCGGTAACGAACGAGACCTCAGCCTGCTAAATAGTTACTCCTACTTTGGTAGGAGGCGAACTTCTTAGAGGGACTATTGGCGTTT
AGCCAATGGAAGTGTGAGGCAATAACAGGTCTGTGATGCCCTTAGATGTTCTGGGCCGCACGCGCGCTACACTGATGCATTCAACGAGCCTAGCCTTGACCGAGAGGTCCGGGTAATCT
TTGAAACTGCATCGTGATGGGGCTAGATTATTGCAATTATTAATCTTCAACGAGGAATGCCTAGTAAGCGTGATTCATCAAATCGCGTTGATTACGTCCCTGCCCTTTGTACACACC

b)

Description	Scientific Name	Max Score	Total Score	Query Cover	E value	Per. Ident	Acc. Len	Accession
Tetraselmis rubens strain RCC133 18S ribosomal RNA gene, partial sequence	Tetraselmis...	2863	2863	100%	0.0	100.00%	1565	KT860871.1
Tetraselmis marina strain CTM 20015 18S ribosomal RNA gene, partial sequence	Tetraselmis...	2863	2863	100%	0.0	100.00%	1636	KT023599.1
Tetraselmis marina strain NZmm1W1 18S ribosomal RNA gene, partial sequence	Tetraselmis...	2857	2857	100%	0.0	99.94%	1744	KY054995.1
Tetraselmis rubens isolate CCAP 66/6 18S ribosomal RNA gene, partial sequence	Tetraselmis...	2837	2837	99%	0.0	99.87%	1603	KX904700.1
Tetraselmis rubens isolate Tetr_R_9_2 18S ribosomal RNA gene, partial sequence	Tetraselmis...	2824	2824	98%	0.0	99.93%	1585	KX904703.1
Tetraselmis rubens isolate Tetr_R_9_3 18S ribosomal RNA gene, partial sequence	Tetraselmis...	2822	2822	98%	0.0	100.00%	1587	KX904704.1
Tetraselmis rubens isolate Tetr_V_2_2 18S ribosomal RNA gene, partial sequence	Tetraselmis...	2817	2817	98%	0.0	99.87%	1586	KX904705.1
Tetraselmis rubens isolate Tetr_Mes_5 18S ribosomal RNA gene, partial sequence	Tetraselmis...	2809	2809	99%	0.0	99.55%	1603	KX904702.1
Tetraselmis rubens isolate Tetr_Mes_17 18S ribosomal RNA gene, partial sequence	Tetraselmis...	2809	2809	99%	0.0	99.55%	1600	KX904701.1
Tetraselmis marina isolate MP0060 small subunit ribosomal RNA gene, partial seq...	Tetraselmis...	2791	2791	100%	0.0	99.16%	1740	KY655019.1
Tetraselmis marina 18S rRNA gene (partial), 5.8S rRNA gene, 28S rRNA gene (par...	Tetraselmis...	2787	2787	100%	0.0	99.10%	5630	HE610131.1
Tetraselmis marina 18S ribosomal RNA gene, partial sequence	Tetraselmis...	2785	2785	100%	0.0	99.10%	1669	KY045847.1

Table S2. Identification of isolate Z3

**Identification of isolate Z3**	
Domain	Eukaryota
Division	Chlorophyta
Class	Chlorodendrophyceae
Order	Chlorodendrales
Famille	Chlorodendraceae
Genus	*Tetraselmis*
Species	sp.

Figure S7. Microscopic pictures of isolate D3

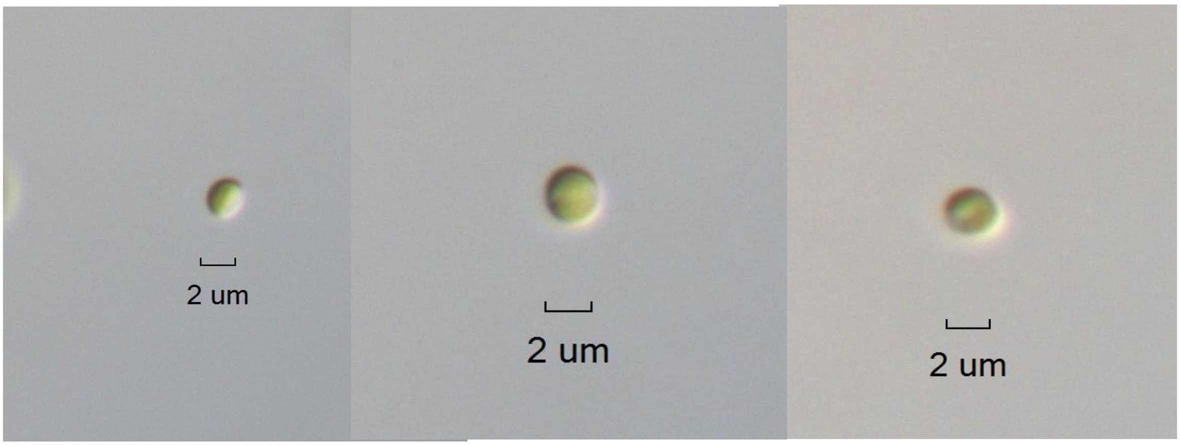

Figure S8. Isolate D3 a) sequence of DNA fragment of 18S rRNA gene; b) BLAST results (NCBI database http://www.ncbi.nlm.nih.gov/)

a)

>Gene 18S rRNA:
AGGCAGCAGGCGCGCAAATTACCCAATCCTGACACAGGGAGGTAGTGACAATAAATAACAATACCGGGCCTTTGGTCTGGTAATTGGAATGAGTACAACCTAAACACCTTAACG
AGGATCAATTGGAGGGCAAGTCTGGTGCCAGCAGCCGCGGTAATTCCAGCTCCAATAGCGTATATTTAAGTTGCTGCAGTTAAAAAGCTCGTAGTTGGATTTCGGGTGGGGCCT
GCCGGTCCGCCGTTTCGGTGTGCACTGGCCNGGGCCCACCTTGTTGCCGGGGACGGGCTCCTGGGCTTCGCTGTCCGGGACCCGGAGTCGGCGAGGTTACTTTGAGTAAATTAG
AGTGTTCAAAGCAGGCCTACGCTCTGAATACATTAGCATGGAATAACACGATAGGACTCTGGCCTATCCTGTTGGTCTGTAGGACCGGAGTAATGATTAAGAGGGACAGTCGGG
GGCATTCGTATTTCATTGTCAGAGGTGAAATTCTTGGATTTATGAAAGACGAACTACTGCGAAAGCATTTGCCAAGGATGTTTTCATTAATCAAGAACGAAAGTTGGGGGCTCGA
AGACGATTAGATACCGTCCTAGTCTCAACCATAAACGATGCCGACTAGGGATCGGCGGGTGTTTTTTTGATGACCCCGCCGGCACCTTATGAGAAATCAAAGTTTTTGGGTTCCG
GGGGGAGTATGGTCGCAAGGCTGAAACTTAAAGGAATTGACGGAAGGGCACCACCAGGCGTGGAGCCTGCGGCTTAATTTGACTCAACACGGGAAAACTTACCAGGTCCAGAC
ATAGTGAGGATTGACAGATTGAGAGCTCTTTCTTGATTCTATGGGTGGTGGTGCATGGCCGTTCTTAGTTGGTGGGTTGCCTTGTCAGGTTGATTCCGGTAACGAACGAGACCTC
AGCCTGCTAACTAGTCACGCGTGCTCCGGCACGCGGCGGACTTCTTAGAGGGACTATTGGCGACTAGCCAATGGAAGCATGAGGCAATAACAGGTCTGTGATGCCCTTAGATGT
TCTGGGCCGCACGCGCGCTACACTGATGCATTCAACGAGCCTATCCTTGGCCGAGAGGTCCGGGTAATCTTTGAATCTGCATCGTGACGGGGATAGATTATTGCAATTATTAATCT
TCAACGAGGAATGCCTAGTAAGCGCAAGTCATCAGCTTGCGTTGATTACGTCCCTGCCC

b)

Description	Scientific Name	Max Score	Total Score	Query Cover	E value	Per. Ident	Acc. Len	Accession
Nannochloropsis oculata strain Xmm23S2 18S ribosomal RNA gene, partial sequence	Nannochlorops...	2209	2209	100%	0.0	99.83%	1708	KY054986.1
Nannochloris sp. KMMCC 161 18S ribosomal RNA gene, partial sequence	Nannochloris s...	2209	2209	100%	0.0	99.83%	1668	JQ315641.1
Nannochloris sp. KMMCC C103 18S ribosomal RNA gene, partial sequence	Nannochloris s...	2209	2209	100%	0.0	99.83%	1669	GQ122342.1
Nannochloris sp. KMMCC C-75 18S ribosomal RNA gene, partial sequence	Nannochloris s...	2209	2209	100%	0.0	99.83%	1669	GQ122337.1
Picochlorum oculatum 18S small subunit ribosomal RNA gene, partial sequence	Nannochlorops...	2209	2209	100%	0.0	99.83%	1692	AY422075.1
Nanochlorum eucaryotum 18S rDNA	Picochlorum eu...	2209	2209	100%	0.0	99.83%	1796	X06425.1
Picochlorum eukaryotum sensu Mainz 1 strain Xmm7W2 18S ribosomal RNA gene, partial sequence	Picochlorum eu...	2204	2204	100%	0.0	99.75%	1711	KY054971.1
Picochlorum eukaryotum sensu Mainz 1 strain Xmm7W1 18S ribosomal RNA gene, partial sequence	Picochlorum eu...	2204	2204	100%	0.0	99.75%	1642	KY054970.1
Picochlorum eukaryotum isolate Xmm37W1 18S ribosomal RNA gene, partial sequence	Picochlorum eu...	2204	2204	100%	0.0	99.75%	1711	KU561201.1
Nannochloropsis oculata isolate Xmm36S2 18S ribosomal RNA gene, partial sequence	Nannochlorops...	2204	2204	100%	0.0	99.75%	1709	KU561163.1
Nannochloris sp. KMMCC 326 18S ribosomal RNA gene, partial sequence	Nannochloris s...	2204	2204	100%	0.0	99.75%	1668	JQ315648.1
Nannochloris sp. KMMCC 861 18S ribosomal RNA gene, partial sequence	Nannochloris s...	2204	2204	100%	0.0	99.75%	1668	JQ315646.1
Nannochloris sp. KMMCC 312 18S ribosomal RNA gene, partial sequence	Nannochloris s...	2204	2204	100%	0.0	99.75%	1668	JQ315643.1
Nannochloris sp. KMMCC 119 18S ribosomal RNA gene, partial sequence	Nannochloris s...	2204	2204	100%	0.0	99.75%	1668	JQ315639.1
Nannochloris sp. KMMCC 118 18S ribosomal RNA gene, partial sequence	Nannochloris s...	2198	2198	100%	0.0	99.67%	1667	JQ315638.1
Nannochloris sp. KMMCC C-166 18S ribosomal RNA gene, partial sequence	Nannochloris s...	2198	2198	100%	0.0	99.67%	1669	GQ122350.1

Figure S9. Isolate D3 a) sequence of DNA fragment of 16S rRNA gene; b) BLAST results (NCBI database http://www.ncbi.nlm.nih.gov/)

a)

Gene 16S:
TCGGCTAACTCTGTGCCAGCAGCCGCGGTAAGACAGAGGATGCAAGCGTTATCCGGATTGATTGGGCGTAAAGCGTCTGTAGGTGGTTTATCCAGTCTTCTGTCAAAGATCAG
GGCTTAACCTTGGATAGGCAGGAGAAACTAATAGACTAGAGTTCGGTAGGGGCAGAGGGAATTCCCGGTGGAGCGGTGAAATGCGTAGAGATCGGGAGGAACACCAAAGG
CGAAAGCACTCTGCTGGGCCGCGACTGACACTGAGAGACGAAAGCGAGGGGAGCAAATGGGATTAGATACCCCAGTAGTCCTCGCCGTAAACGATGGATACTAGGTGTTGG
GTGATATTACATTCAGTACCGTAGCTAACGCGTGAAGTATCCCGCCTGGGGAGTATGCTCGCAAGAGTGAAACTCAAAGGAATTGACGGGGGCCCGCACAAGCGGTGGAGCA
TGTGGTTTAATTCGATGCAACGCGAAGAACCTTACCAGGGCTTGACATGCCACTTTTTTCTTGAAAAAGAAAGTTCCCGAGTGGACACAGGTGGTGCATGGCTGTCGTCAGCTC
GTGTCTTGAGATGTTGGGTTAAGTCCCGCAACGAGCGCAACCCTTATTTTGTGTTGCTATTTTTAGGAAACTCAAAAGACTGCCGGTGATAAGCCGGAGGAAGGTGAGGATGA
CGTCAAGTCAGCATGCCCCTTACGCCCTGGGCCACACACGTGCTACAATGGCTGGGACAAAGAGATGCAACCCCGCGAGGGCAAGCCAACCTC

b)

Description	Scientific Name	Max Score	Total Score	Query Cover	E value	Per. Ident	Acc. Len	Accession
Picochlorum sp. RCC475 chloroplast partial 16S rRNA gene, strain RCC 475	Picochlorum sp....	1395	1395	98%	0.0	99.87%	791	LN735427.3
Picochlorum sp. 'soloecismus' strain DOE 101 chloroplast, complete genome	Picochlorum sp. '...	1393	1393	100%	0.0	99.48%	72741	MG552671.1
Chlorella sp. UMPCCC 1312 16S ribosomal RNA gene, partial sequence; chloroplast	Chlorella sp. UM...	1393	1393	100%	0.0	99.48%	1114	KT438808.1
Chlorella sp. UMPCCC 1231 16S ribosomal RNA gene, partial sequence; plastid	Chlorella sp. UM...	1393	1393	100%	0.0	99.48%	1114	KM218898.1
Chlorella sp. UMPCCC 1222 16S ribosomal RNA gene, partial sequence; plastid	Chlorella sp. UM...	1393	1393	100%	0.0	99.48%	1113	KM218896.1
Uncultured organism clone L73_ML_035 small subunit ribosomal RNA gene, partial sequence; chloroplast	uncultured organ...	1336	1336	95%	0.0	99.46%	842	FJ353941.1
Nannochloris sp. 5-RCC9 16S ribosomal RNA gene, partial sequence; plastid	Nannochloris sp....	1247	1247	100%	0.0	95.97%	898	AY702134.1
Chlorella sp. UMPCCC 1309 16S ribosomal RNA gene, partial sequence; chloroplast	Chlorella sp. UM...	1245	1245	100%	0.0	95.97%	1061	KT438813.1

Figure S10. Isolate D3 a) sequence of DNA fragment of LSU gene; b) BLAST results (NCBI database http://www.ncbi.nlm.nih.gov/)

a)

Gene LSU:
AAGCATATCAATAAGCGGAGGAAAAGAAACTAACCAGGATTCCCCTAGTAACGGCGAGCGAACCGGGAAGAGCCCAACGTGAAAATCTCCAGCCTTYGGCTGGCGAATTGTA
GTCTAGAGACGCGTGCTCTGTGCCTGYTCCGGCCCAAGTCCCCTGGAAAGGGGCGTCCGAGAGGGTGAGAACCCCGTGGGGCCGGTGCTTGGGCACCTCACGAGCCGCCGTC
GTCGAGTCGGGTTGCTTGGGAATGCAGCCCAAAGCAGGTGGTAAATCCCATCTAAGGCTAAATACCGACGGGAGACCGATAGCGAACAAGTACCGTGAGGGAAAGATGAAAA
GCACTTTGAAAAGAGAGTTAAAAAGTACTTGAAATTGTTGAGAGGGAAGAGATGGGGTCGCCGCGGGTGCGCCCAGGCACACGCCCGCCCTGGTGGCTGGTTGAATGCACTG
GGCGCTGGTCAGCATGGGTTGGTGGGGCGGGATACCCCCGGGGCTTTGTGCCCCGGTCGTTGCCGCCCCGTCGACCGAGGAACTGCAGGGCGCTCTCCGAGTCCTTTTGGAAC
TGCGCCCTCGAGATGCTGGCGG

b)

Description	Scientific Name	Max Score	Total Score	Query Cover	E value	Per. Ident	Acc. Len	Accession
Chloroidium saccharophilum voucher MZCH 10155 18S ribosomal RNA gene, partial sequence; internal transcrib...	Chloroidium sacc...	676	676	100%	0.0	87.71%	1629	KX525236.1
Chlorella vulgaris 28S ribosomal RNA gene, partial sequence	Chlorella vulgaris	665	665	100%	0.0	87.39%	630	KM272627.1
Micractinium sp. KNUA032 28S ribosomal RNA gene, partial sequence	Micractinium sp. ...	662	662	99%	0.0	87.33%	612	KM243323.1
Chlorella sp. 'South China Sea' 18S ribosomal RNA gene, partial sequence; internal transcribed spacer 1, 5.8S ri...	Chlorella sp. 'So...	662	662	100%	0.0	87.22%	1411	KF998566.1
Dictyosphaerium sp. HN362 18S ribosomal RNA gene, partial sequence; internal transcribed spacer 1, 5.8S ribos...	Dictyosphaerium ...	660	660	99%	0.0	87.20%	3461	MF664526.1
Dictyosphaerium sp. GL29 small subunit ribosomal RNA gene, partial sequence; internal transcribed spacer 1, 5....	Dictyosphaerium ...	660	660	99%	0.0	87.20%	3005	MF664498.1
Dictyosphaerium ehrenbergianum strain GL26_2 small subunit ribosomal RNA gene, partial sequence; internal tra...	Dictyosphaerium ...	658	658	99%	0.0	87.16%	3019	MF664495.1
Dictyosphaerium sp. HN361 18S ribosomal RNA gene, partial sequence; internal transcribed spacer 1, 5.8S ribos...	Dictyosphaerium ...	654	654	99%	0.0	87.14%	3462	MF664525.1
Dictyosphaerium sp. HND35 18S ribosomal RNA gene, partial sequence; internal transcribed spacer 1, 5.8S ribos...	Dictyosphaerium ...	654	654	99%	0.0	87.14%	3459	MF664524.1

Table S3. Identification of isolate D3

**Identification of isolate D3**	
Domain	Eukaryota
Division	Chlorophyta
Class	Trebouxiophyceae
Order	Chlorellales
Famille	Chlorellaceae
Genus	*Picochlorum*
Species	sp.

Figure S11. Microscopic pictures of isolate C6

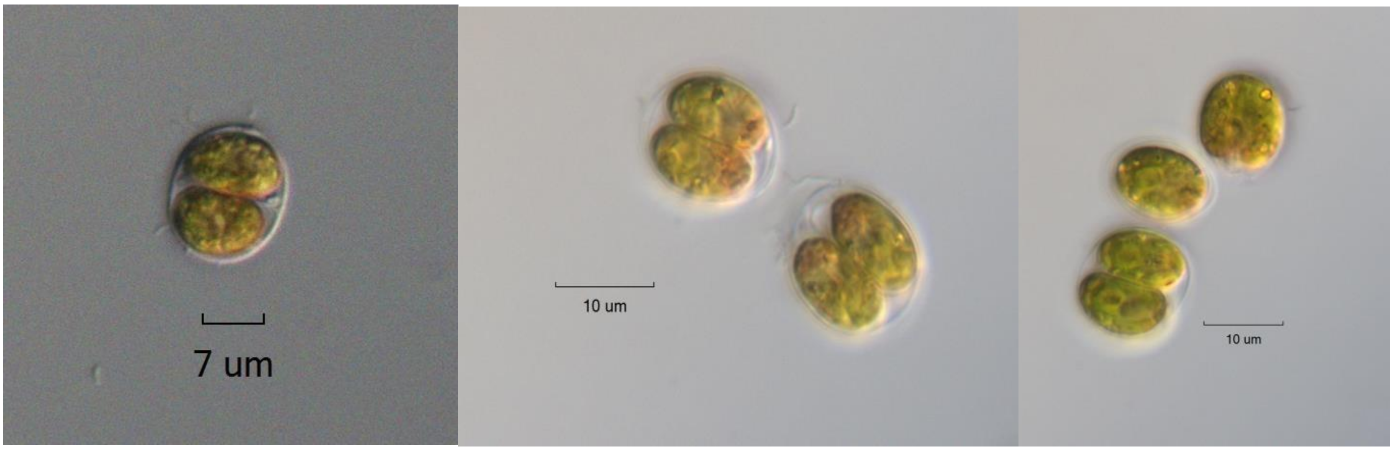

Figure S12. Isolate C6 a) sequence of DNA fragment of 18S rRNA gene; b) BLAST results (NCBI database http://www.ncbi.nlm.nih.gov/)

a)

>Gene 18S rRNA:
ATGCATGTCTAAGTATAAACTGCTTATACTGTGAAACTGCGAATGGCTCATTAAATCAGTTATAGTTTATTTGATGGTACCTACTACTCGGATAACCGTAGTAATTCTAGAGCTAATA
CGTGCGTAAATCCCGACTTCTGGAAGGGACGTATTTATTAGATTTAAGGCCAACCGAGCTCTGCTCGTCTCTTGGTGAATCATGATAACTTCACGAATCGCATGGCCCCTGCGCCGG
CGATGTTTCATTCAAATTTCTGCCCTATCAATTGGCGATGGTAGGATAGAGGCCTACCATGGTGTTAACGGGTGACGGAGGATTAGGGTTCGATTCCGGAGAGGGAGCCTGAGAA
ACGGCTACCACATCCAAGGAAGGCAGCAGGCGCGCAAATTACCCAATCCTGATACAGGGAGGTAGTGACAATAAATAACAATACCGGGCTTTTCAAGTCTGGTAATTGGAATGAG
TACAATCTAAATCCCTTAACGAGGATCCATTGGAGGGCAAGTCTGGTGCCAGCAGCCGCGGTAATTCCAGCTCCAATAGCGTATATTTAAGTTGTTGCAGTTAAAAAGCTCGTAGT
TGGATTTCGGATGGGACTTGCCGGTCCGTCGTTGCGATGTGCACTGGCCAGTCCTATCTTGTTGTCGGGGACTAGCTCTTGGACTTAACTGCCCGGGACTAGGAGCTGACGAGGTT
ACTTTGAGTAAATTAGAGTGTTCAAAGCAAGCCTACGCTCTGAATACATTAGCATGGAATAACACGATAGGACTCTGGCTTATCCTGTTGGTCTGTGAGACCAGAGTAATGATTAA
GAGGGACAGTCGGGGACATTCGTATTTCATTGTCAGAGGTGAAATTCTTGGATTTATGAAAGACGAACTTCTGCGAAAGCATTTGTCAAGGATGTTTTCATTAATCAAGAACGAAA
GTTGGGGGCTCGAAGACGATTAGATACCGTCCTAGTCTCAACCATAAACGATGCCGACTAGGGATTGGCAGACGTTTTTTTGATGACTCTGCCAGCACCTTATGAGAAATCAAAGT
TTTTGGGTTCCGGGGGGAGTATGGTCGCAAGGCTGAAACTTAAAGGAATTGACGGAAGGGCACCACCAGGCGTGGAGCCTGCGGCTTAATTTGACTCAACACGGGAAAACTTAC
CAGGTCCAGACATAGTGAGGATTGACAGATTGAGAGCTCTTTCTTGATTCTATGGGTGGTGGTGCATGGCCGTTCTTAGTTGGTGGGTTGCCTTGTCAGGTTGATTCCGGTAACGA
ACGAGACCTCAGCCTGCTAAATAGTTACTCCTACTTTGGTAGGAGGCGAACTTCTTAGAGGGACTATTGGCGTTTAGCCAATGGAAGTGTGAGGCAATAACAGGTCTGTGATGCCC
TTAGATGTTCTGGGCCGCACGCGCGCTACACTGATGCATTCAACGAGCCTAGCCTTGACCGAGAGGTCCGGGTAATCTTTGAAACTGCATCGTGATGGGGCTAGATTATTGCAATT
ATTAATCTTCAACGAGGAATGCCTAGTAAGCGTGATTCATCAAATCGCGTTGATTACGTCCCTGCCCTTTGTACACACCGCCCGTCGCTCCTACCGATTGAGTGTGTTGGTGAGGAG
TTCGGATTGGCTCTTAGTGGTGGTTCGCCACCACCTAGAGCTGAGAAGTTCTCCAAACCGCCCCACT

b)

Description	Scientific Name	Max Score	Total Score	Query Cover	E value	Per. Ident	Acc. Len	Accession
Tetraselmis marina strain NZmm1W1 18S ribosomal RNA gene, partial sequence	Tetraselmis ma...	3088	3088	99%	0.0	99.64%	1744	KY054995.1
Tetraselmis marina isolate MP0060 small subunit ribosomal RNA gene, partial sequence	Tetraselmis ma...	2994	2994	99%	0.0	98.64%	1740	KY655019.1
Tetraselmis marina 18S rRNA gene (partial), 5.8S rRNA gene, 28S rRNA gene (partial), ITS1 and ITS...	Tetraselmis ma...	2992	2992	100%	0.0	98.58%	5630	HE610131.1
Tetraselmis marina strain CTM 20015 18S ribosomal RNA gene, partial sequence	Tetraselmis ma...	2977	2977	96%	0.0	99.51%	1636	KT023599.1
Tetraselmis rubens isolate Tetr_Mes_5 18S ribosomal RNA gene, partial sequence	Tetraselmis rub...	2955	2955	94%	0.0	99.94%	1603	KX904702.1
Tetraselmis rubens isolate Tetr_Mes_17 18S ribosomal RNA gene, partial sequence	Tetraselmis rub...	2950	2950	94%	0.0	99.94%	1600	KX904701.1
Prasinophyte symbiont of radiolarian 257/331/333 from cf. Spongodrymus 257 18S ribosomal RNA g...	prasinophyte s...	2950	2950	100%	0.0	98.17%	1793	AF166379.1
Tetraselmis marina 18S ribosomal RNA gene, partial sequence	Tetraselmis ma...	2948	2948	98%	0.0	98.56%	1669	KY045847.1
Uncultured marine eukaryote clone SA2_1B6 18S ribosomal RNA gene, partial sequence	uncultured mar...	2940	2940	99%	0.0	98.11%	1840	EF527133.1
Tetraselmis sp. MBIC11125 gene for 18S rRNA, partial sequence	Tetraselmis sp....	2940	2940	100%	0.0	98.05%	1752	AB058392.1
Prasinophyte symbiont of radiolarian 257/331/333 from cf. Spongodrymus 333 18S ribosomal RNA g...	prasinophyte s...	2940	2940	100%	0.0	97.99%	1793	AF166381.1
Tetraselmis sp. AL-FL22 small subunit ribosomal RNA gene, partial sequence	Tetraselmis sp....	2939	2939	100%	0.0	98.05%	1755	MH055454.1
Prasinophyte symbiont of radiolarian 257/331/333 from cf. Spongodrymus 331 18S ribosomal RNA g...	prasinophyte s...	2939	2939	100%	0.0	98.05%	1793	AF166380.1

Figure S13. Isolate C6 a) sequence of DNA fragment of LSU gene; b) BLAST results (NCBI database http://www.ncbi.nlm.nih.gov/)

a)

Gene LSU:
TGAACGCAGCTCCGAAGGCAAAGCGTTCTTAAACCTCGATCGACGAAGCGGCATAGGCAGTGTAACAATCACTACTTTTGTCCCGCTAGGCCAATCCATGCTGACCAGCTTCTAGCA
CTGAGGGGCAGTCCTGTTAGAGACTACATACAGCCTAGAAGCACCCACGCCTCTAATACACTTCCCCCTCAACAATTTCAAGCACTTTTTAACTCTCTTTTCAAAGGCCTTTTCATCTTT
CCCTCGCGGTACTTGTTCGCTATCGGTCTCCTACCGGTATTTAGCCTTAGAAGGGATGTACCTCCCAATTAGGGCTGGATTCTCAACCAACCCGACTCTGCGAAAGCATCTCGTGAAG
AGGCTAAGACCAAGGATGACGGGGTTCTCACCCTCTCTGACGTCCCCTTCCAGGGAACTTAATCCAGGTCCGCCTCAGAGGTTGCTTCTATAGACTACAATTCGGCAGACAAAGCCT
GCAGATTTTCAAGTTGGGCTTATCCCGTTTCACTCGCCGTTACTAAGGGAATCCTAGTTAGTTTCTTTTCCTCCGCTTATTGATATGCTTAAA

b)

Description	Scientific Name	Max Score	Total Score	Query Cover	E value	Per. Ident	Acc. Len	Accession
Tetraselmis suecica isolate MK1 28S ribosomal RNA gene, partial sequence	Tetraselmis sue...	931	931	99%	0.0	96.46%	850	KC415759.1
Tetraselmis marina isolate MP0060 large subunit ribosomal RNA gene, partial sequence	Tetraselmis marina	931	931	99%	0.0	96.46%	1238	KY655021.1
Tetraselmis marina 18S rRNA gene (partial), 5.8S rRNA gene, 28S rRNA gene (partial), ITS1 and ITS2, strain...	Tetraselmis marina	931	931	99%	0.0	96.46%	5630	HE610131.1
Tetraselmis marina isolate MP0018 large subunit ribosomal RNA gene, partial sequence	Tetraselmis marina	928	928	99%	0.0	96.45%	807	KY655018.1
Tetraselmis sp. SMS19 small subunit ribosomal RNA gene, partial sequence; internal transcribed spacer 1, 5...	Tetraselmis sp....	880	880	99%	0.0	94.85%	3575	MT489380.1
Symbiodinium sp. strain SMS5 strain SMS19 internal transcribed spacer 2 and large subunit ribosomal RNA ...	Symbiodinium s...	880	880	99%	0.0	94.85%	1435	MT489354.1
Tetraselmis striata 18S rRNA gene (partial), 5.8S rRNA gene, 28S rRNA gene (partial), ITS1 and ITS2, strain ...	Tetraselmis striata	857	857	99%	0.0	94.15%	4027	HE610129.1
Tetraselmis chuii gene for 26S rRNA, partial sequence, strain: NIES-2432	Tetraselmis chuii	832	832	91%	0.0	95.74%	2844	AB491617.1

Table S4. Identification of isolate C6

**Identification of isolate C6**	
Domain	Eukaryota
Division	Chlorophyta
Class	Chlorodendrophyceae
Order	Chlorodendrales
Famille	Chlorodendraceae
Genus	*Tetraselmis*
Species	sp.

Figure S14. Microscopic pictures of isolate S5

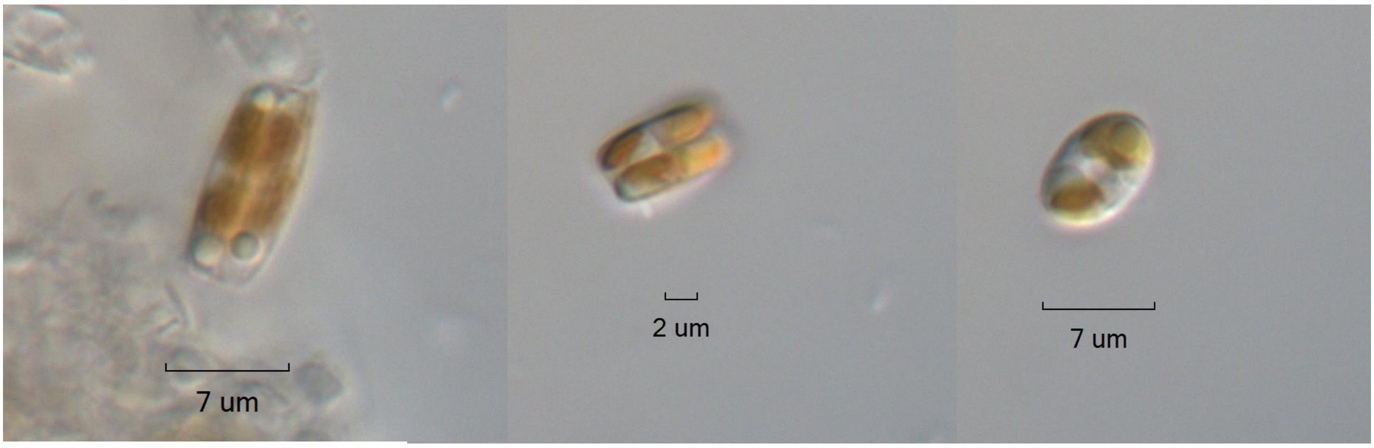

Figure S15. Isolate S5 a) sequence of DNA fragment of 18S rRNA gene; b) BLAST results (NCBI database http://www.ncbi.nlm.nih.gov/)

a)

>Gene 18S rRNA:
GATTAAGCCATGCATGTCTAAGTATAAATCTCTTACTTTGAAACTGCGAACGGCTCATTATATCAGTTATAGTTTATTTGATAGTCCCTTACTACTTGGATAACCGTAGTAATTCTAGAG
CTAATACATGCGTCAATACCCTTCTGGGGTAGTATTTATTAGATTGAAACCAACCCCTTCGGGGTGATGTGGTGATTCATAATAAGCTTGCGGATCGCATGCCTTACGGCGGCGATGG
ATCATTCAAGTTTCTGCCCTATCAGCTTTGGATGGTAGGGTATTGGCCTACCATGGCTTTAACGGGTAACGGAAATTAGGGTTTGATTCCGGAGAGGGAGCCTGAGAGACGGCTAC
CACATCCAAGGAAGGCAGCAGGCGCGTAAATTACCCAATCCTGACACAGGGAGGTAGTGACAATAAATAACAATGCCGGGCCTTTGTAGGTCTGGCAATTGGAATGAGAACAATTT
AAACCCCTTATCGAGTATCAATTGGAGGGCAAGTCTGGTGCCAGCAGTCGCGGTAATTCCAGCTCCAATAGCGTATATTAAAGTTGTTGCAGTTAAAAAGCTCGTAGTTGGATTTGTG
GCTGTCGGTCGCGGCCTATCACTCAGTGTTAGTGCCTGCAGCCGTCGCCATCCTTGGGTGGAGCCTGTGTGGCATTAGGTTGTCGTGCAGGGGATGCCCATCGTTTACTGTGAGAAA
ATTAGAGTGTTCAAAGCAGGCTTATGCCGTTGAATATGTTAGCATGGAATAATAAGATAGGACCTCGGTACTATTTTGTTGGTTTGCGCACCGAGGTAATGATTAATAGGGACAGTTG
GGGGTATTCGTATTTCATTGTCAGAGGTGAAATTCTTGGATTTCTGAAAGACGAACTACTGCGAAAGCATTTACCAAGGATGTTTTCATTAATCAAGAACGAAAGTTAGGGGATCGAA
GATGATTAGATACCATCGTAGTCTTAACCATAAACTATGCCGACAAGGGATTGGTGGAGTTCCGTTTTGTCTCCATCAGCACCTTATGAGAAATCACAAGTCTTTGGGTTCCGGGGGG
AGTATGGTCGCAAGGCTGAAACTTAAAGAAATTGACGGAAGGGCACCACCAGGAGTGGAGCCTGCGGCTTAATTTGACTCAACACGGGAAAACTTACCAGGTCCAGACATAGTGAG
GATTGACAGATTGAGAGCTCTTTCTTGATTCTATGGGTGGTGGTGCATGGCCGTTCTTAGTTGGTGGAGTGATTTGTCTGGTTAATTCCGTTAACGAACGAGACCCCTGCCTGCTAAA
TAGCTTGCCGAGTGAGTTTTCACTGGGTGAAGCTTCTTAGAGGGACGTGCATTCTATTAGATGCAGGAAGATAGGGGCAATAACAGGTCTGTGATGCCCTTAGATGTCCTGGGCCGC
ACGCGCGCTACACTGATGCATTCAACGAGTCCTTCCTTGGCCGAGAGGCCTGGGTAATCTTTTGAACGTGCATCGTGATAGGGATAGATTATTGCAATTATTAATCTTGAACGAGGAA
TTCCTAGTAAACGCAGTTCATCAAACTGCATTGATTACGTCCCTGCCCTTTGTACACACCGCCCGTCGCACCTACCGATTGAATGGT

b)

Description	Scientific Name	Max Score	Total Score	Query Cover	E value	Per. Ident	Acc. Len	Accession
Uncultured marine eukaryote clone I-7-MC660-OTU-28 18S ribosomal RNA gene, p…	uncultured …	2802	2802	100%	0.0	97.96%	1786	KC771178.1
Nitzschia sp. SZCZCH658 18S ribosomal RNA gene, partial sequence	Nitzschia sp…	2796	2796	100%	0.0	97.90%	1733	KT943651.1
Nitzschia traheaformis isolate SZCZCH972 18S ribosomal RNA gene, partial sequen…	Nitzschia tr…	2796	2796	100%	0.0	97.90%	1731	KT943644.1
Nitzschia sp. AnM0026 small subunit ribosomal RNA gene, partial sequence	Nitzschia sp…	2796	2796	100%	0.0	97.90%	1711	EU090031.1
Nitzschia dubiiformis gene for 18S rRNA, partial sequence, strain: s0311	Nitzschia d…	2791	2791	100%	0.0	97.83%	1780	AB430616.1
Nitzschia navis-varingica isolate MC5366 small subunit ribosomal RNA gene, partial …	Nitzschia n…	2787	2787	100%	0.0	97.78%	1710	MT239347.1
Nitzschia sp. (in: Bacillariophyta) strain KSA2015-11 acute rect-ED-1 small subunit ri…	Nitzschia sp…	2774	2774	100%	0.0	97.65%	1724	MH063490.1
Nitzschia sp. (in: Bacillariophyta) strain KSA2015-23 penn-B1 small subunit ribosom…	Nitzschia sp…	2769	2769	100%	0.0	97.59%	1739	MH063486.1
Nitzschia dubia strain TA37 18S ribosomal RNA gene, partial sequence	Nitzschia d…	2765	2765	98%	0.0	97.93%	1680	KY320381.1
Psammodictyon pustulatum strain KSA2015-38 FORAM pandur-FO1 small subunit r…	Psammodic…	2758	2758	100%	0.0	97.46%	1745	MH063502.1
Nitzschia linearis culture TCC:386 small subunit ribosomal RNA gene, partial seque…	Nitzschia lin…	2758	2758	100%	0.0	97.46%	1785	MN696696.1
Nitzschia linearis culture TCC:387 small subunit ribosomal RNA gene, partial seque…	Nitzschia lin…	2752	2752	100%	0.0	97.40%	1790	MN696697.1
Nitzschia dubiiformis strain SH366 18S ribosomal RNA gene, partial sequence	Nitzschia d…	2747	2747	99%	0.0	97.57%	1659	KY320382.1
Nitzschia sp. (in: Bacillariophyta) strain Nate Site 1 elongate peanut Nitz small subu…	Nitzschia sp…	2743	2743	98%	0.0	97.68%	1623	MH040324.1
[illegible]	[illegible]	[illegible]	[illegible]	[illegible]	[illegible]	[illegible]	[illegible]	[illegible]

Table S5. Identification of isolate S5

**Identification of isolate S5**	
Domain	Eukaryota
Division	Bacillariophyta
Class	Bacillariophyceae
Order	Bacillariales
Famille	Bacillariaceae
Genus	*Nitzschia* or *Psammodictyon*
Species	sp.

Figure S16. Microscopic pictures of isolate C1

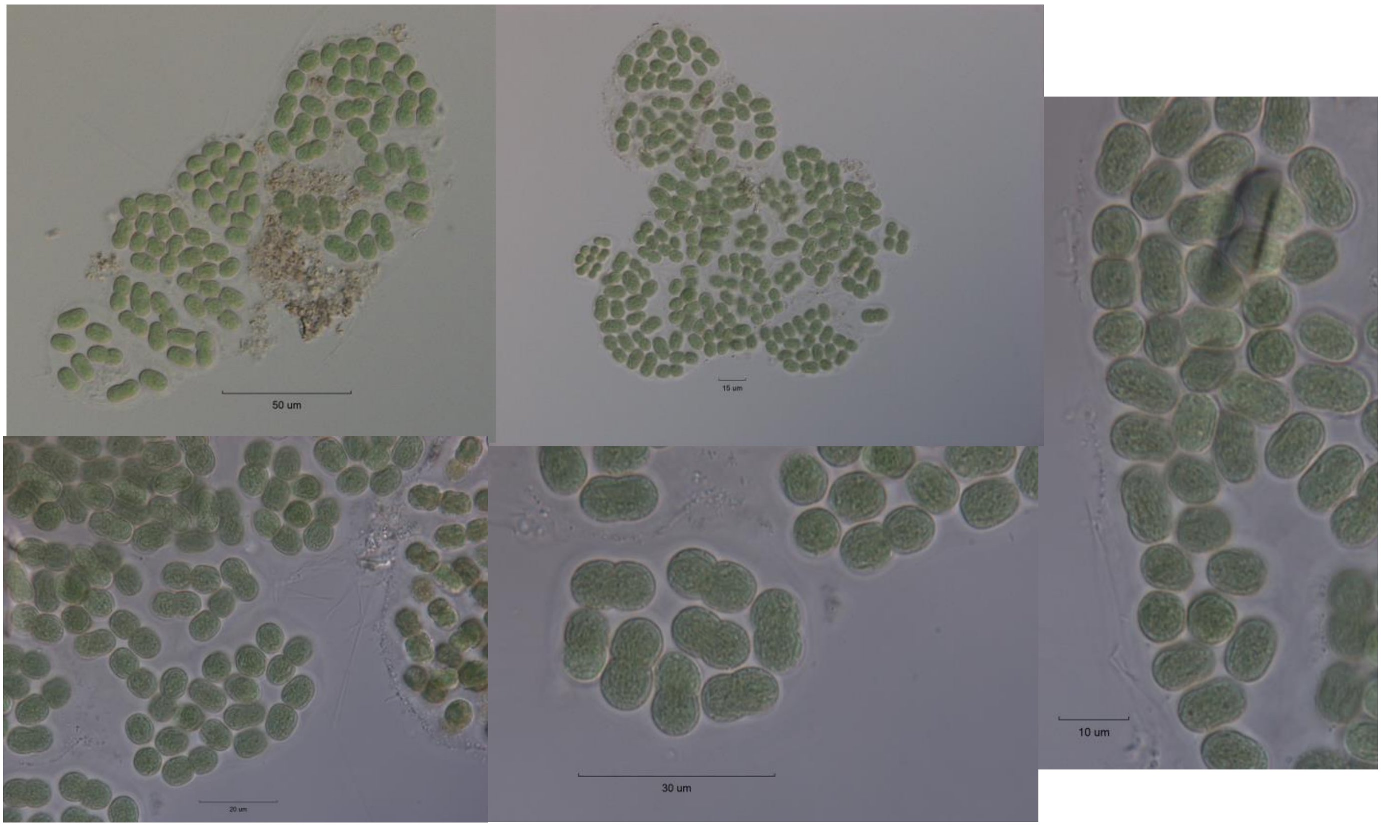

Figure S17. Isolate C1 a) sequence of DNA fragment of 16S rRNA gene; b) BLAST results (NCBI database http://www.ncbi.nlm.nih.gov/)

a)

Consensus 16S:
CGCTGGCGGCGTGCTTAACACATGCAAGTCGAACGAAGCCTTCGGGCTGAGTGGCGGACGGGTGAGTAACGCGTGAGAATCTACCTTCAAGATGGGGACAACCGTTGGAAACG
ACGGCTAATCCCCAATAAGCCGAAAGGTGAAAAGGGTTTCCGCTTGAAGAGGAGCTCGCGTCCGATTAGTTAGTTGGTGGGGTAAGAGCCTACCAAGGCAGCGATCGGTAGCT
GGTCTGAGAGGATGAGCAGCCACACTGGGACTGAGACACGGCCCAGACTCCTACGGGAGGCAGCAGTGGGGAATTTTCCGCAATGGGCGAAAGCCTGACGGAGCAACGCCGC
GTGTGGGAAGAAGGCTTTTGGGCTGTCAACCACTTTTCTCAGGGAAGAAGTTCTGACGGTACCTGAGGAATCAGCCTCGGCTAACTCCGTGCCAGCAGCCGCGGTAATACGGAG
GAGGCAAGCGTTATCCGGAATTATTGGGCGTAAAGCGTCCGCAGGGGGCACATCAAGTCTGCTGTCAAAGGTCGGAGCTTAACTCCGGTTCGGCAGTGGAAACTGGGGTGCTA
GAGGGCGACAGGGGTAGAGGGAATTCCCAGTGTAGCGGTGAAATGCGTAGAGATTGGGAAGAACACCGGTGGCGAAAGCGCTCTACTGGGTCGCACCTGACCCTCAGGGACG
AAAGCTAAGGTAGCGAAAGGGATTAGATACCCCTGTAGTCTTAGCCGTAAACGATGGATACTAGGCGTGGTTTGTATCGACCCAAGCCGTGCCGAAGCTAACGCGTTAAGTATC
CCGCCTGGGGAGTACGCACGCAAGTGTGAAACTCAAAGGAATTGACGGGGGCCCGCACAAGCGGTGGAGGATGTGGTTTAATTCGATGCAACGCGAAGAACCTTACCAGGGCT
TGACATCCCGCGAATCTTGCTGAAAGGTGAGAGTGCCTTCGGGAACGCGGAGACAGGTGGTGCATGGCTGTCGTCAGCTCGTGTCGTGAGATGTTGGGGTTAAGTCCCGCAAC
GAGCGCAACCCTCGTCCTTAGTTGCCAGCATTAAGTTGGGCACTCTAGGGAGACTGCCGGTGACAAACCGGAGGAAGGTGAGGATGACGTCAAGTCAGCATGCCCCTTATGCTC
TGGGCAACACACGTCCTACAATGGTCGGGACAAAGGGGAGCAAGCTCGCGAGAGTAAGCGAATCCCATCAAACTCGGCCTCAGTTCAGATTGCAGGCTGCAACTCGCCTGCATG
AAAGGAGGAATCGCTAGTAATCGCCGGTCAGCATACGGNCGGTGAATTCGTTCCCGGGCCTTGTACACACCGCCCGTCACACCATGGAAGCTGGCCAAGCCCGAAGTCGTTACC
C

b)

Description	Scientific Name	Max Score	Total Score	Query Cover	E value	Per. Ident	Acc. Len	Accession
Euhalothece sp. 16S rRNA gene, strain MPI 95AH13	Euhalothece sp....	2346	2346	99%	0.0	97.94%	1399	AJ000710.1
Euhalothece sp. BDU 130911 16S ribosomal RNA gene, partial sequence	Euhalothece sp....	2316	2316	100%	0.0	97.43%	1376	KM350247.1
Euhalothece sp. BDU 130192 16S ribosomal RNA gene, partial sequence	Euhalothece sp....	2316	2316	100%	0.0	97.43%	1379	KF498709.1
Euhalothece sp. 16S rRNA gene, strain MPI 96N304	Euhalothece sp....	2298	2298	100%	0.0	97.21%	1452	AJ000713.1
Euhalothece sp. 16S rRNA gene, strain MPI 96N303	Euhalothece sp....	2298	2298	100%	0.0	97.21%	1452	AJ000712.1
Halothece sp. PCC 7418 chromosome, complete genome	Halothece sp. P...	2287	6850	100%	0.0	97.06%	4179170	CP003945.1
Cyanothece sp. GSL007 16S ribosomal RNA gene, partial sequence	Cyanothece sp....	2287	2287	100%	0.0	97.06%	1410	FJ546715.1
Cyanothece sp. 113 16S ribosomal RNA gene, partial sequence	Cyanothece sp....	2287	2287	100%	0.0	97.06%	1455	DQ243689.1
Euhalothece sp. 16S rRNA gene, strain MPI 95AH10	Euhalothece sp....	2283	2283	100%	0.0	96.99%	1436	AJ000709.1
Halothece sp. PCC 7418 16S ribosomal RNA gene, partial sequence	Halothece sp. P...	2281	2281	100%	0.0	96.99%	1442	AF296872.1
Cyanothece sp. 115 16S ribosomal RNA gene, partial sequence	Cyanothece sp....	2281	2281	100%	0.0	96.99%	1455	DQ243690.1
Cyanothece sp. 109 16S ribosomal RNA gene, partial sequence	Cyanothece sp....	2281	2281	100%	0.0	96.99%	1455	DQ243688.1

Table S6. Identification of isolate C1

Identification of isolate C1	
Domain	Prokaryota
Division	Cyanobacteria
Class	Oscillatoriophyceae
Order	Chroococales
Famille	Aphanothecacae
Genus	*Euhalothece*
Species	sp.
